# The topoisomerase 3α zinc-finger domain T1 of *Arabidopsis thaliana* is required for targeting the enzyme activity to Holliday junction-like DNA repair intermediates

**DOI:** 10.1371/journal.pgen.1007674

**Published:** 2018-09-17

**Authors:** Annika Dorn, Sarah Röhrig, Kristin Papp, Susan Schröpfer, Frank Hartung, Alexander Knoll, Holger Puchta

**Affiliations:** Botanical Institute, Molecular Biology and Biochemistry, Karlsruhe Institute of Technology, Karlsruhe, Germany; The University of North Carolina at Chapel Hill, UNITED STATES

## Abstract

Topoisomerase 3α, a class I topoisomerase, consists of a TOPRIM domain, an active centre and a variable number of zinc-finger domains (ZFDs) at the C-terminus, in multicellular organisms. Whereas the functions of the TOPRIM domain and the active centre are known, the specific role of the ZFDs is still obscure. In contrast to mammals where a knockout of TOP3α leads to lethality, we found that CRISPR/Cas induced mutants in Arabidopsis are viable but show growth retardation and meiotic defects, which can be reversed by the expression of the complete protein. However, complementation with AtTOP3α missing either the TOPRIM-domain or carrying a mutation of the catalytic tyrosine of the active centre leads to embryo lethality. Surprisingly, this phenotype can be overcome by the simultaneous removal of the ZFDs from the protein. In combination with a mutation of the nuclease AtMUS81, the *TOP3α* knockout proved to be also embryo lethal. Here, expression of TOP3α without ZFDs, and in particular without the conserved ZFD T1, leads to only a partly complementation in root growth—in contrast to the complete protein, that restores root length to *mus81-1* mutant level. Expressing the *E*. *coli* resolvase *RusA* in this background, which is able to process Holliday junction (HJ)-like recombination intermediates, we could rescue this root growth defect. Considering all these results, we conclude that the ZFD T1 is specifically required for targeting the topoisomerase activity to HJ like recombination intermediates to enable their processing. In the case of an inactivated enzyme, this leads to cell death due to the masking of these intermediates, hindering their resolution by MUS81.

## Author summary

DNA Topoisomerases are essential for transcription, DNA repair and DNA replication due to their function to break and change the topological state of DNA molecules. Topoisomerase 3α is especially important for DNA repair as it is able to process different DNA recombination and repair intermediates. The protein can be divided into three different functional domains, the TOPRIM domain, the active centre and the zinc-finger domains (ZFDs). Whereas the first two domains are essential for the basic functions of DNA binding and breaking, the role of the ZFDs was elusive until now. By deletion analysis and complementation studies in the model plant *Arabidopsis thaliana*, we were now able to show that ZFDs are required to target the topoisomerase to specific DNA structures that arise during the repair of aberrant DNA replication intermediates, the Holliday Junctions (HJs). We were able to demonstrate, by expressing a bacterial HJ resolvase in plant cells, that Topoisomerase 3α can only process these intermediates when the ZFDs are part of the protein. However, in the case of a non-functional topoisomerase, the presence of the ZFDs leads to masking of these HJs so that they cannot be processed by other plant nucleases, leading to cell death.

## Introduction

The processing of DNA recombination intermediates like Holliday junctions (HJs) is of utmost importance for all cells to prevent chromosome breakage and ultimately cell death. While the concerted activity of nucleases in the resolution pathway resolves double Holliday junctions (dHJs) to crossover and non-crossover products, a second pathway called dissolution exclusively leads to non-crossover products and relies on the coordinated action of a RecQ-helicase (BLM in mammals, Sgs1 in yeast) and a type IA topoisomerase (TOP3α in higher, Top3 in lower eukaryotes) [1–3]. Supported by the structural protein RMI1, they form the RTR-complex [4]. The RTR-complex is highly conserved in all eukaryotes. Mutations in the human RTR helicase BLM are associated with Bloom’s syndrome, a hereditary disease that is characterised by dwarfism, increased predisposition for cancer and an increased rate of sister chromatid exchanges on the cellular level [5–7]. RecQ-helicases and type IA topoisomerases were shown to interact in the dissolution of dHJs [3,8]. Thereby, the mechanism of dissolution is divided into two steps. First, the two junctions of the dHJ migrate together via the RecQ-helicases branch migration activity to form a hemicatenane intermediate. Then only the topoisomerase dissolves this structure through its action as a decatenase [3,9–11]. This reaction is reversible and does not require ATP. The process is mediated by a transesterification in which a nucleophilic (also referred to as catalytic) tyrosine residue forms a transient 5’ phosphotyrosine ssDNA intermediate [12,13]. Thus, a single strand break is introduced into the DNA and the transfer of the second DNA strand through this gap can change the topology of the DNA. The structural protein RMI1 (RecQ-mediated genome instability) was identified as the third complex partner in which the protein does not possess any catalytic function but stimulates and stabilises the complex [10,14]. In humans, RMI2 exists as an additional complex partner which interacts with RMI1 but no homolog is present in yeast [15–17]. In multicellular eukaryotes, two homologs of Top3 are present: TOP3α and TOP3β, although only TOP3α could be identified as the functional homolog to ScTop3. In mammals TOP3α is essential as *top3α* mutants die in an early stage of embryogenesis [18]. Furthermore, essential functions for TOP3α could be shown in *C*. *elegans* and *D*. *melanogaster* [19,20]. The lethal phenotypes of *top3α* mutants complicate the analysis of protein functions *in vivo*. In contrast to this, *top3β* mutants do not show noticeable phenotypes in *Drosophila melanogaster* or DT40 cells. Mouse *top3β* mutants feature a shortened lifespan, aneuploidy and defects in DNA damage response but no similar phenotypes were shown in other organisms [21–23]. A role for TOP3β as RNA-topoisomerase in humans was postulated, where it is involved in the expression of mRNAs that are important for neuronal development and mental health [24,25].

In *Arabidopsis thaliana*, the presence of all components of the RTR-complex was demonstrated and a complex consisting of RECQ4A, TOP3α, RMI1 and RMI2 was confirmed *in vivo* [26,27]. As RecQ helicase is in the complex, RECQ4A could be identified as the functional homolog of HsBLM and ScSgs1 [28,29]. For all RTR-complex partners, the characteristic mutant phenotypes of hyperrecombination and sensitivity against genotoxins were shown. Additionally a special role for RMI1 and TOP3α in meiosis could be demonstrated, as mutant lines exhibit meiotic defects absent in *recq4A* mutants [17,30–33]. Furthermore a function for RMI2 in the preservation of 45S rDNA repeats was recently unveiled [33]. Interestingly, both currently available T-DNA insertion mutant lines for *TOP3α* in *Arabidopsis* show distinct unique phenotypes. The *top3A-1* mutant is lethal. Seedlings are able to germinate, but cotyledons are deformed and no roots are formed so the plants die shortly after germination [29]. In contrast to this lethal phenotype, the second T-DNA insertion line *top3A-2* is viable but shows strong growth defects like dwarfism and fasciation. So far, *top3A-1* was considered as null mutant whereas *top3A-2* was postulated to show a hypomorphic phenotype. The viable phenotype of *top3A-2* enabled detailed analyses of the mutant. All typical RTR phenotypes such as hyperrecombination and an increased sensitivity against MMS and cisplatin were confirmed but the mutant is additionally sensitive against the topoisomerase I inhibitor camptothecin (CPT). On the cellular level, mitotic aberrations were demonstrated that were absent in mutants of *RMI1* and *RECQ4A*, thus indicating a unique role for TOP3α in mitosis [31]. Furthermore, TOP3α and RMI1 possess essential functions in meiosis. Homozygous mutant lines of both genes are completely sterile and show distinct meiotic defects resulting in an abortion of meiosis [31,32]. Dual functions for AtTOP3α and AtRMI1 in meiosis were identified: Both factors are not only important for the resolution of recombination intermediates but also for the suppression of class II crossovers [27,34]. AtTOP3α consists of 926 aa, divided into six domains. The N-terminus consists of a TOPRIM domain that is conserved in type IA and type II topoisomerases, as well as primases [11,35]. Crystal analyses in human TOP3α confirmed the binding of a bivalent metal ion by the TOPRIM domain, and that it is part of the proteins active centre [36]. The central domain harbours the catalytic tyrosine that is essential for the topoisomerase activity of the protein [37]. The C-terminus consists of four zinc-finger domains. Zinc finger domain T1 is typical for type IA topoisomerases and can be detected in bacterial Topoisomerase 1 and 3, but also in TOP3α homologs of higher eukaryotes. Zinc-finger domain GRF is characterised through a motif of the three-conserved aa GRF in its centre. Moreover, in AtTOP3α, two types of CCHC zinc-finger domains (ZnFCCHC1/2) can be found that are characterised by the aa sequence C-X2-C-X4-H-X4-C [38]. While only zinc-finger T1 is conserved in all TOP3α homologs of higher eukaryotes, the arrangement of the other zinc-finger domains varies between different organisms. Little is known about the functions of these zinc-finger domains.

In parallel to the RTR-complex, the resolvase MUS81 can process DNA-intermediates like dHJs, D-loops and blocked replication forks and acts in a complex together with EME1 (Mms4 in yeast) [39–43]. A simultaneous deficiency in both pathways was shown to be lethal in different eukaryotes, including Arabidopsis [41,44–46]. This synthetic lethality can be complemented by an additional defect in homologous recombination, thus indicating the accumulation of unprocessed, toxic HR intermediates as cause for the observed lethality [45,47,48]. In previous experiments, it was additionally shown that the overexpression of the bacterial resolvase EcRusA can resolve persisting Holliday junctions in yeast and human, leading to a rescue from toxic recombination intermediates [39,49,50].

To clarify the phenotype of *top3α* null mutants in Arabidopsis we performed a Cas9-mediated mutagenesis of the *TOP3α* gene. Our results demonstrate that *top3α* null mutants in Arabidopsis are viable, which is unique for higher eukaryotes. Furthermore, we performed complementation experiments in *top3α* mutants using both the full-length *TOP3α* gene and recombinant *TOP3α* variants in which domains were either deleted or altered. These approaches demonstrated that an impaired catalytic activity of TOP3α unexpectedly leads to embryo lethality, which can be rescued by an additional deletion of the C-terminal zinc-finger domains of the protein. We could also show that these zinc-finger domains are dispensable for most functions of TOP3α. However, in the absence of MUS81, zinc-finger domain T1 is required for the repair of replication associated DNA damage in the root meristem. This deficiency can be overcome by the expression of the bacterial resolvase RusA. This indicates a role for this zinc-finger in targeting the topoisomerase activity to specific DNA repair intermediates like Holliday junctions.

## Results

### CRISPR/Cas induced null mutants of *TOP3α* are viable in Arabidopsis

**Fig 1:**
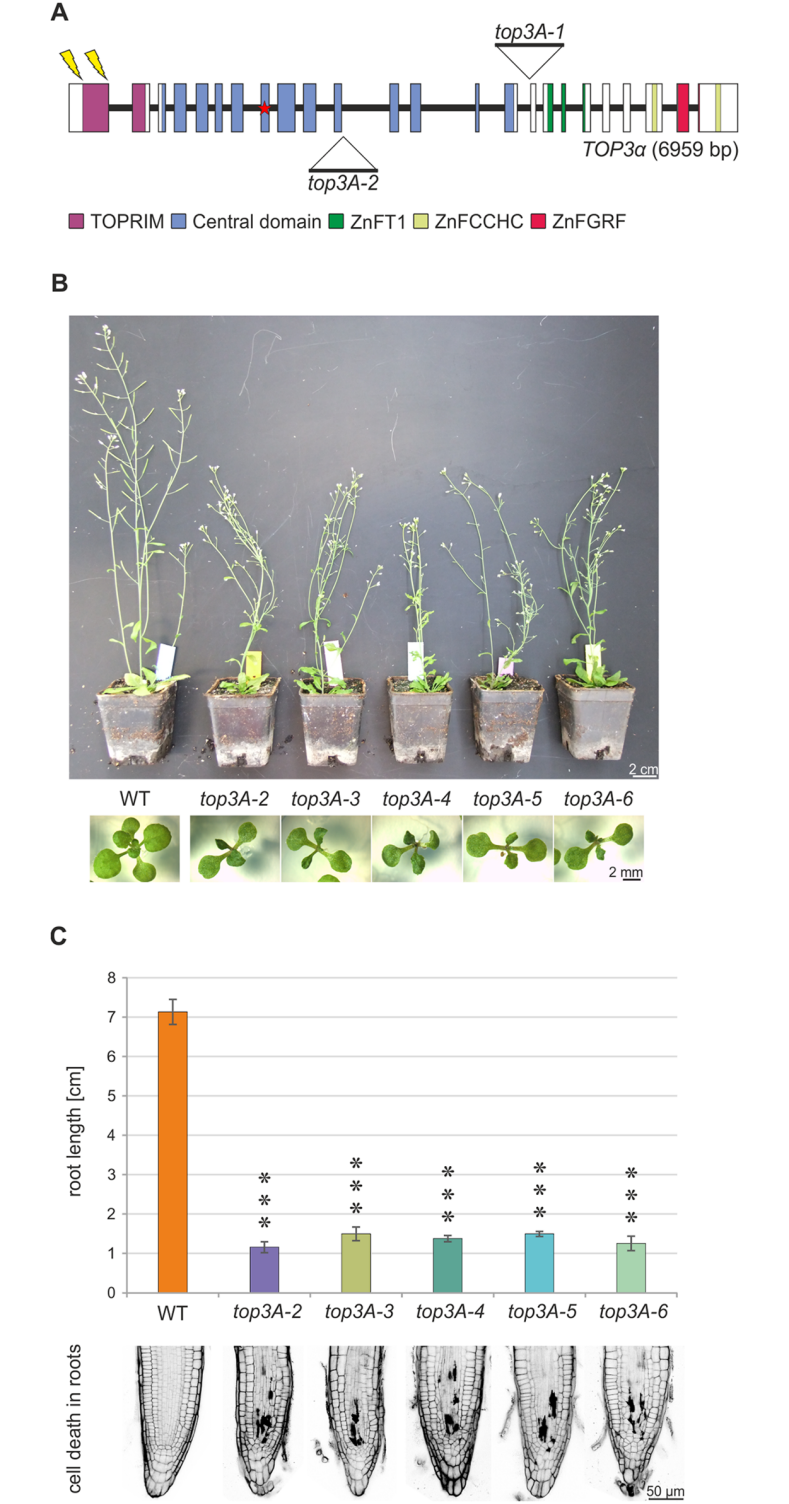
At*top3α* mutants exhibit a viable but growth restricted phenotype. (A) AtTOP3α gene and domain structure. The *TOP3α* gene comprises 6959 bp in 24 exons (boxes). T-DNA insertions in *top3A-1* and *top3A-2* mutant lines are located in intron 15 and 11, respectively. For CRISPR/Cas9 mediated mutagenesis, target sequences in exon 1 were used (bolts). The TOP3α protein harbours six domains, a TOPRIM domain, the central domain with the catalytic tyrosine (star) and four C-terminal zinc-finger domains. (B) At*top3α* mutant phenotypes. Two-week-old seedlings and six-week-old plants of the T-DNA insertion mutant *top3A-2* and four CRISPR/Cas9 induced mutant lines *top3A-3*, *top3A-4*, *top3A-5* and *top3A-6* are compared to wild type (WT) plants of the same age. All mutant lines depict an identical growth restricted phenotype. (C) Root length and cell death analysis in *top3α* mutant lines. Root length of ten days old *top3α* mutant lines compared to the wild type (WT) was determined in three independent assays and mean values with standard deviation (error bars) were calculated. All *top3α* mutant lines show a significant reduction of root length compared to WT plants. Thereby, root length among individual mutant lines was comparably reduced. Statistical differences to the WT were calculated using a two-tailed t-test with unequal variances: *** p < 0.001. Dead cells in the root meristem were visualized with propidium iodide staining of five days old plant roots. While no cell death was visible in WT roots, *top3α* mutant lines exhibited a vast number of dead cells.

In *Arabidopsis thaliana*, several *top3α* mutant lines with different phenotypes have been reported so far (S1 Fig). Whilst the *top3A-1* mutant line exhibits a lethal phenotype with barely germinating, deformed seedlings, the *top3A-2* line is viable, but exhibits various growth defects [29,31]. Three further *top3α* mutant lines were reported to have no growth defects but enhanced meiotic crossover formation [27,34]. Until now, due to the similar phenotype in mammals, the lethal *top3A-1* mutant line was considered the null mutant, while a hypomorphic phenotype was postulated for the other mutants. To redefine the null mutant phenotype, we performed a Cas9-mediated mutagenesis of At*TOP3α* as previously established in our group [51]. Two independent approaches for mutagenesis in exon 1 of *TOP3α* were performed, the first used a target sequence starting 11 nucleotides after the start codon (protospacer 5’-CCGGCGATGTCGCGACGAGG-3’), the second target was chosen after nucleotide 271 (protospacer 5’-GGCAGATCTGTACCAAGCTC-3’, Fig 1A). For each approach, two different mutant lines could be established, named *top3A-3* and 4 and *top3A-5* and *6*, respectively. The mutations in these lines lead to a frameshift in the open reading frame of the *TOP3α* gene and thereby generated premature stop codons. Sanger sequencing of cDNA (S2 Fig) verified mutations on mRNA level. Surprisingly, all obtained mutant lines were viable and demonstrated a growth-restricted phenotype with dwarfism, fasciated organs and deformed leaves identical to the previously described *top3A-2* mutant line (Fig 1B). For the quantification of growth defects, root length was determined. DNA damages that lead to replication fork blocks can obstruct cell division in highly proliferating tissues. As the growth of plant roots is based on cell divisions in the root meristem, they are a suitable system for the quantification of replication-associated DNA damages [52]. Root length of 10 days old plants was determined in the obtained mutant lines, in comparison to the *top3A-2* line and wild type (WT) plants (Fig 1C). All *top3α* mutant lines exhibited comparable (1.2–1.5 cm) and, compared to WT plants (7.1 cm), significantly reduced root lengths. The root length defect was based on an increased cell death in the root meristem, visualized by propidium iodide staining of dead cells in five days old seedlings (Fig 1C). All *top3α* mutant lines exhibited a high amount of dead cells in the root meristems, while no dead cells were present in WT roots.

The newly obtained *top3α* mutant lines were further analysed. A prominent feature of RTR mutants is their hyperrecombination phenotype. The reporter construct IC9C, for the analysis of interchromosomal recombination events, was introduced into the *top3A-4* and *top3A-6* mutants by cross-breeding. Restoration of the β-glucuronidase gene in the IC9C reporter is only possible by recombination with the sister chromatid or homologous chromosome [53]. Both mutant lines confirmed the elevated recombination rate of *top3A-2* mutants compared to WT plants (S3 Fig). Furthermore, the hypersensitive phenotype of *top3A-2* mutants against the intrastrand crosslinker cisplatin, the methylating agent methylmethanesulfonate (MMS) and the topoisomerase I inhibitor camptothecin (CPT) could be confirmed in the *top3A-4* and *top3A-6* mutant lines (S3 Fig). Additionally, an increased sensitivity against the crosslinker mitomycin C (MMC) could be demonstrated in all analysed *top3α* mutant lines, hinting to a so far uncharacterized involvement of AtTOP3α in interstrand CL repair (S3 Fig). A further characteristic of *top3A-2* mutants is their fertility defect. All *top3α* mutant lines show a completely sterile phenotype identical to the *top3A-2* mutant line (S4 Fig). Detailed analyses of DAPI stained meiocytes in the *top3A-4* and *top3A-6* mutant lines further confirmed the unique meiotic defects observed in *top3A-2* (S4 Fig). While the beginning of meiosis appears normal with full synapsis in pachytene, from the onset of diplotene extensive chromosome bridges and fragmentation could be observed. These defects ultimately lead to an arrest after meiosis I, therefore no meiocytes of later stages than dyads could be identified.

These results demonstrate that in contrast to mammals a complete loss of TOP3α in Arabidopsis is viable and results in a phenotype similar to the one described for the *top3A-2* mutant.

### Functional domain analysis of AtTOP3α

**Fig 2:**
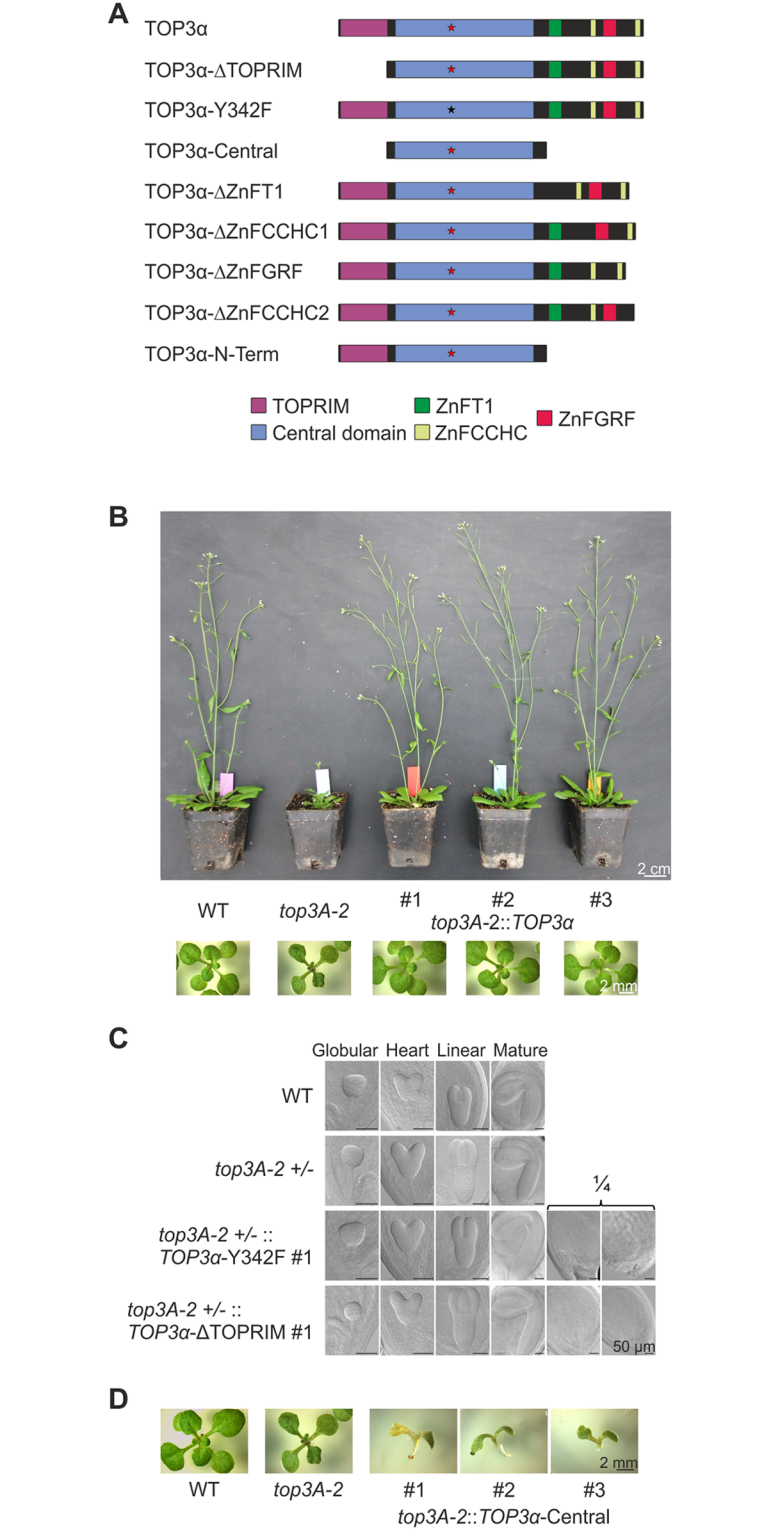
Complementation analyses in *top3α* mutants. (A) Schematic structure of TOP3α protein variants used for complementation analyses. In TOP3α-ΔTOPRIM the TOPRIM domain was deleted. The protein variant TOP3α-Y342F harbours an amino acid substitution, replacing the catalytic tyrosine (red star) with a phenylalanine (black star). In TOP3α-Central both the TOPRIM domain and the C-terminus including all zinc-finger domains were deleted. In the protein variants TOP3α-ΔZnFT1/ΔZnFCCHC1/ΔZnFGRF/ΔZnFCCHC2 the individual zinc-finger domains were deleted. The variant TOP3α-N-Term consists of the N-terminus of TOP3α without C-terminal zinc-finger domains. (B) Full complementation of *top3A-2* growth phenotype by expression of *TOP3α*. Two-week-old seedlings and six-week-old plants from three individual *top3A-2*::*TOP3α* complementation lines are compared to *top3A-2* mutants and wild type (WT) plants. The characteristic growth defects of *top3A-2* could be fully complemented by expression of *TOP3α* in all three complementation lines, leading to a growth phenotype indistinguishable to WT plants. (C) Embryo development in *top3A-2* +/- ::*TOP3α*-Y342F/ΔTOPRIM. Depicted are representative embryos of exemplary *top3A-2* +/- ::*TOP3α*-Y342F and *top3A-2* +/- ::*TOP3α*-ΔTOPRIM lines compared to wild type (WT) and *top3A-2* +/- embryos. All lines showed a complete embryo development leading to mature embryos. In heterozygous *top3A-2* complementation lines, a Χ^2^ test confirmed a ratio of ¼ seeds with lacking or deformed embryos, corresponding to the amount of homozygous *top3A-2* mutants containing the complementation construct. (D) Growth phenotype of *top3A-2*::*TOP3α*-Central lines. Two-week-old plantlets of three individual *top3A-2*::*TOP3α*-Central complementation lines are compared to *top3A-2* mutants and wild type (WT) plants. While *top3A-2* mutant lines exhibit characteristic growth defects with dark, deformed leaves, expression of *TOP3α*-Central in this line leads to an enhanced growth defect. Plants feature only the cotyledons that are deformed and no roots are formed.

For a functional analysis of the protein domains in AtTOP3α, complementation studies were performed. Therefore, complementation constructs with deleted or altered domains were transformed into *top3A-2* and *top3A-6* mutant lines (Fig 2A). All constructs were cloned from gDNA and were under the control of the natural *TOP3α* promoter and terminator. The construct *TOP3α*-ΔTOPRIM lacks the N-terminal TOPRIM domain. In the construct *TOP3α*-Y342F, the catalytic tyrosine was substituted with phenylalanine by introducing a point mutation. The constructs *TOP3α*-ΔZnFT1/ΔZnFCCHC1/ΔZnFGRF/ΔZnFCCHC2 harbour a deletion of the corresponding zinc-finger domain. The construct *TOP3α*-N-Term consists of the TOP3α N-terminus without C-terminal zinc-finger domains. In the construct *TOP3α*-Central, both TOPRIM domain and C-terminal zinc-fingers were deleted, so the remaining protein only consists of the central domain. As a positive control, the construct *TOP3α*, harbouring the natural *TOP3α* gene without modifications was used. All constructs were transformed into the *top3A-2* mutant line and the wild type. Additionally, the constructs *TOP3α*, *TOP3α*-ΔTOPRIM, *TOP3α*-Y342F, *TOP3α*-N-Term and *TOP3α*-Central were transformed into the *top3A-6* mutant line. Wild type plants containing the different complementation constructs were viable, did not show any growth anomalies and were fully fertile (S5 Fig). For the verification of our results, for every transformation approach, at least three independent homozygous, single locus lines were established. As *top3α* mutant lines show a completely sterile phenotype, transformation was performed using plants heterozygous for the *top3α* mutation. The *top3A-2* complementation lines harbouring the full-length *TOP3α* gene showed a growth phenotype indistinguishable from wild type plants (Fig 2B). For a detailed analysis, the complementation lines were tested regarding the characteristic defects of the mutant line. Introduction of the full-length *TOP3α* construct in the *top3A-2* mutant lead to a complete complementation of fertility, root length, hyperrecombination, as well as sensitivity against cisplatin, CPT, MMS and MMC (S6 Fig). All complementation constructs transformed into the *top3A-6* mutant line showed exactly the same phenotypes as with the *top3A-2* mutant (S7 Fig; S1 Table), indicating that there is no interference of putative truncated proteins of the respective mutants with the TOP3α protein variants.

The N-terminal part of AtTOP3α consists of the TOPRIM domain followed by the central domain. For human TOP3α, the binding of a divalent metal ion by the TOPRIM domain was demonstrated, and also that the TOPRIM domain participates in the active centre of the protein [36]. The catalytic function of topoisomerases is mediated by a nucleophilic tyrosine. Replacement of the catalytic tyrosine by phenylalanine in topoisomerases was shown to inhibit enzymatic activity [54,55]. To analyse the function of the TOPRIM domain and the catalytic tyrosine in AtTOP3α, two complementation constructs were used in which the TOPRIM domain was deleted and the catalytic tyrosine was substituted with a phenylalanine, respectively. Both constructs were transformed into the *top3A-2* and *top3A-6* mutant lines. However, no homozygous mutants containing either complementation construct could be identified. Thus, embryo development in siliques of heterozygous *top3α* mutants containing either of the complementation constructs, was determined. Fig 2C depicts the embryo development in exemplarily chosen *top3A-2* complementation lines. Embryo development in wild type seeds proceeds over certain microscopically distinguishable stages. Starting from the sphere shaped globular stage, the cotyledonary primordia develop in heart stage, followed by cell expansion and cell division in the embryo first leading to linear stage and ultimately resulting in a mature embryo with apparent cotyledons and root meristem [56]. In all lines, correct embryo development including mature embryos could be observed, but a striking accumulation of seeds with lacking or deformed embryos was determined in all complementation lines. This implied an unexpected embryo lethal phenotype for homozygous *top3α* mutants containing either complementation construct. In this case, following mendelian genetics, ¼ of all seeds should be affected. For the verification of this hypothesis, a Χ^2^-test was performed, testing if the amount of seeds with lacking or deformed embryo matches ¼ of all seeds (S1 and S2 Tables). While the ratio of seeds with lacking or deformed embryos corresponded to ¼ in all analysed complementation lines, this was not the case in the wild type and heterozygous mutant controls. Thus, the expression of AtTOP3α with the deletion of the TOPRIM domain or mutation of the catalytic tyrosine, lead to embryo lethality in mutant Arabidopsis plants. This was extremely surprising, as it indicated that in the absence of a functional protein, the expressed modified protein induces a severe negative effect on plant cells.

### Additional deletion of TOP3α zinc-finger domains rescues the embryo lethal phenotype of TOPRIM deletion

To further define the nature of this negative effect, we produced a construct in which in addition to the deletion of the TOPRIM domain, the C-terminus of TOP3α including all C-terminal zinc-finger domains, was deleted resulting in the construct *TOP3α*-Central consisting solely of the TOP3α central domain. The construct was transformed into *top3A-2* and *top3A-6* mutants and for each approach, three complementation lines were established. To our surprise, complementation lines harbouring the *TOP3α*-Central construct were able to germinate again, although exhibiting an enhanced growth defect compared to the *top3α* mutant lines (Fig 2D, S7 Fig). Complementation lines were able to germinate but cotyledons were deformed, no roots were formed and plants died afterwards. These results imply a rescue of the embryo lethal phenotype of *TOP3α*-ΔTOPRIM deletion lines by an additional deletion of the C-terminal zinc-finger domains.

### The TOP3α zinc-finger domains are expendable for most protein functions

**Fig 3:**
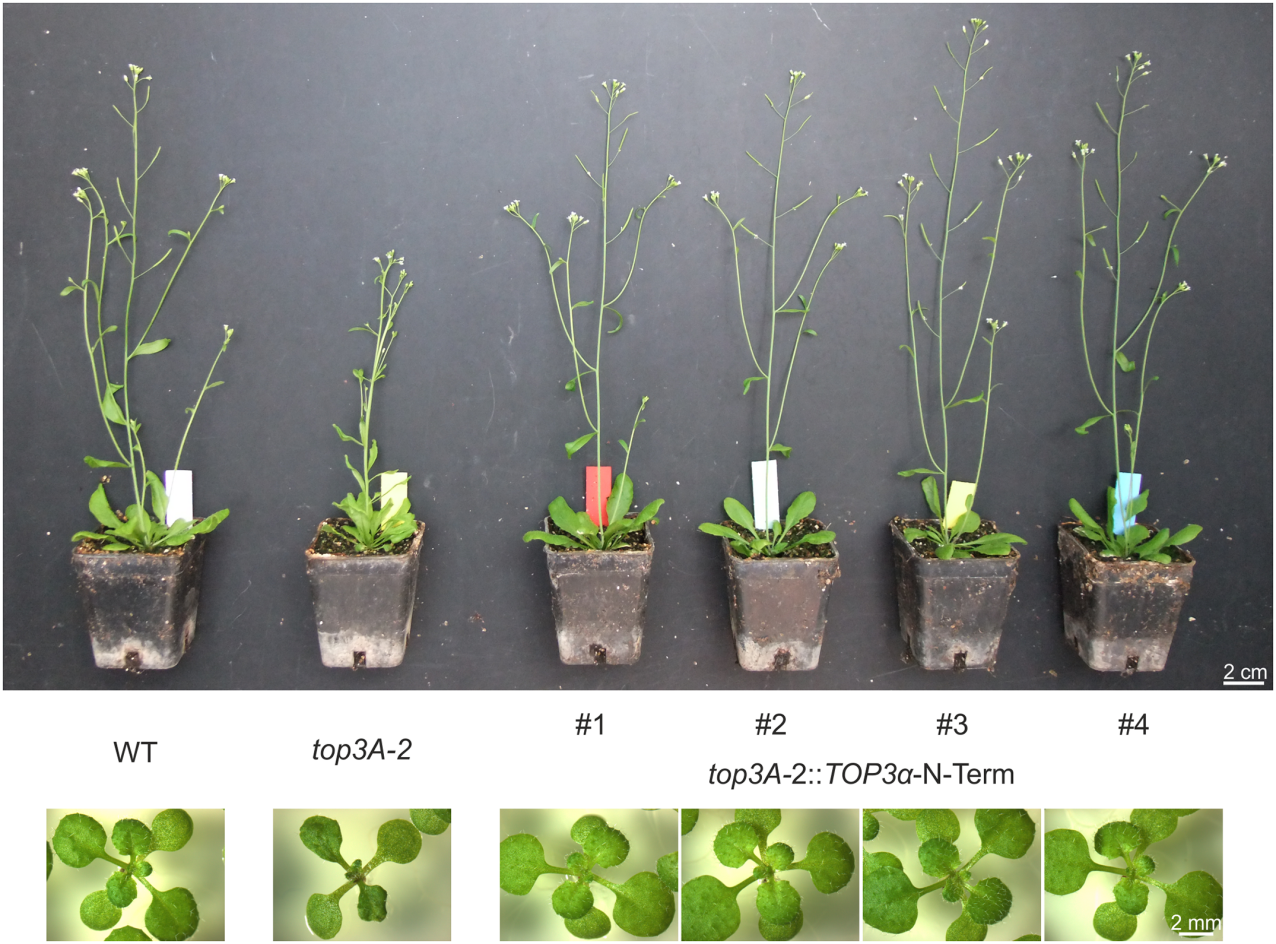
Growth phenotype of *top3A-2*::*TOP3α*-N-Term lines. Two-week-old plantlets and five-week-old plants of four individual *top3A-2*::*TOP3α*-N-Term complementation lines are compared to *top3A-2* mutants and wild type (WT) plants. The growth defects of *top3A-2* mutant plants could be complemented completely by expression of *TOP3α*-N-Term leading to plants indistinguishable from the WT.

As our previous analysis revealed the biological importance of the zinc-finger domains, we wanted to address their role in detail and used the complementation construct *TOP3α*-N-Term, harbouring the N-terminal part of TOP3α without C-terminal zinc-finger domains. The construct was transformed into *top3A-2* and *top3A-6* mutant lines and at least three independent homozygous, single locus lines of each were established. Remarkably, although almost one third of the TOP3α protein is missing, complementation lines containing the *TOP3α*-N-Term construct exhibited a growth phenotype indistinguishable from the wild type and were completely fertile (Fig 3, S7 Fig). For a detailed phenotypical analysis, four independent *top3A-2*::TOP3α-N-Term complementation lines were examined. Indeed, the typical growth phenotype with reduced root length could be reversed in all complementation lines (S8 Fig). Fertility analyses further revealed a restoration of full fertility in the complementation lines and the typical hyperrecombination phenotype of *top3A-2* mutant lines, as well as hypersensitivity against MMS, cisplatin, CPT and MMC, could be rescued by the expression of *TOP3α*-N-Term (S8 Fig). Thus, the C-terminal zinc-finger domains of TOP3α are expendable for most functions of the protein.

### TOP3α zinc-finger domains are not involved in transcriptional control

Zinc-finger domains were first identified in the *Xenopus laevis* transcription factor IIIA and they are common to a wide range of transcription factors [57,58]. Thus, an obvious possibility for a function of TOP3α zinc-finger domains besides DNA repair would be an involvement in transcriptional control. To test this hypothesis, we analysed gene expression via RNA-seq in a *top3A-2*::*TOP3α*-N-Term line in comparison to the original *top3A-2* mutant line and wild type plants. The analysis was conducted in biological triplicates and differentially expressed genes (DEGs) between different genotypes were calculated. Differential expression was assigned with a FDR value < 0.05. While *top3A-2* mutants exhibited 719 statistical significant DEGs in comparison to wild type plants, only 4 genes were found to be differentially expressed in *top3A-2*::*TOP3α*-N-Term compared to the wild type (S3 Table). Among those genes was *TOP3α* itself (up-regulated 4.5x), *QQS* (qua-quine starch, At3G30720, up-regulated 3.6x), *MRD1* (mto1 responding down, At1G53480, up-regulated 158x) and *HEI10* (At1G53490, up-regulated 6x). As HEI10 is a known meiotic DNA recombination factor, we investigated the up-regulation observed with this gene. However, further analysis of the *HEI10* cDNA 5’ end showed that the transcription start site was 1025 bp further downstream than originally annotated; leading to a misalignment of reads (S2 Method). These reads could be designated to the gene directly next to *HEI10*, *MRD1*, which showed a strong induction in our RNA-seq analysis. Indeed, qRT-PCR analysis of mRNA expression from both genes confirmed no significant up-regulation of *HEI10* (S9 Fig). As only an extremely small number of genes was found to be differentially expressed, we concluded that the C-terminal zinc-finger domains in TOP3α are not involved in transcriptional control.

### The zinc-finger domain T1 is involved in replication-associated DNA repair in the root meristem parallel to MUS81

As we found no indication of a function for TOP3α zinc-finger domains in transcriptional regulation, we questioned as to whether they might contribute to TOP3α function in helping to process specific DNA repair intermediates. Because the sole deletion of TOP3α zinc-finger domains did not have detectable consequences in our previous repair assays, we studied the effects of missing TOP3α zinc-finger domains in the absence of a further repair pathway acting on recombination intermediates. Typically, proteins of the RTR complex and the resolvase MUS81 act in parallel in the resolution of recombination intermediates. While the RTR-complex is involved in the dissolution pathway, MUS81 acts as an endonuclease in the resolution of DNA intermediates like blocked replication forks or D-loops [41,42]. Double mutants of the RTR-complex RecQ helicase RECQ4A and MUS81 were shown by us to be synthetically lethal in Arabidopsis [46]. For the analysis of the relationship between MUS81 and TOP3α, we aimed to generate a *top3A-2 mus81-1* double mutant by cross-breeding. As we were unable to identify homozygous double mutants, we analysed embryo development in siliques of double mutants heterozygous for *top3A-2* (S10 Fig). Statistical analysis by performing a Χ^2^ test confirmed that ¼ of all seeds in *top3A-2* +/- *mus81-1* -/- double mutants contained deformed or missing embryos (S4 Table). As this ratio is in accordance with mendelian segregation leading to ¼ homozygous double mutants, we propose that *top3A-2 mus81-1* double mutants are embryo lethal.

**Fig 4:**
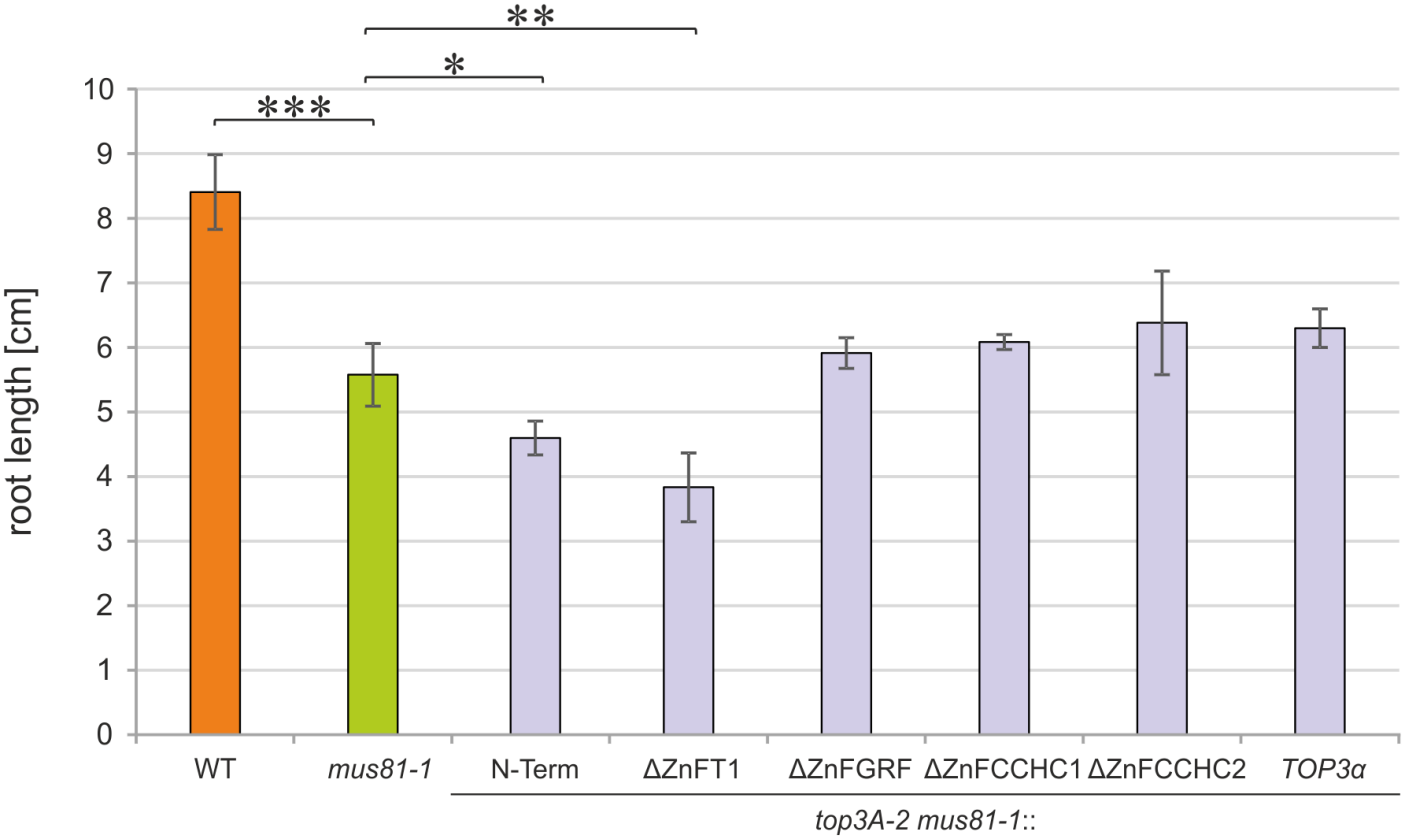
Root length analysis of *top3A-2* complementation lines in *mus81-1* mutant background. Root length of ten-day-old *top3A-2* complementation lines in *mus81-1* background, wild type (WT) plants and *mus81-1* mutants was determined in four independent assays and mean values with standard deviation (error bars) were calculated. In *mus81-1* mutants, a significantly reduced root length compared to the WT was determined. Compared to *mus81-1*, both *top3A-2 mus81-1*::*TOP3α*-N-Term and *top3A-2 mus81-1*::*TOP3α*-ΔZnFT1 exhibit a significantly reduced root length while this was not the case for the further complementation lines. Statistical differences were calculated using a two-tailed t-test with unequal variances: * p < 0.05, ** p < 0.01, *** p < 0.001.

To investigate if certain zinc-finger domains are necessary for the function of AtTOP3α in the absence of MUS81, *top3A-2 mus81-1* double mutants harbouring the *TOP3α* complementation constructs *TOP3α*-N-Term, *TOP3α*-ΔZnFT1, *TOP3α*-ΔZnFGRF, *TOP3α*-ΔZnFCCHC1, *TOP3α*-ΔZnFCCHC2 and the full-length *TOP3α* construct as control, were generated by cross-breeding of established *top3A-2* complementation lines with *mus81-1*. For the quantification of possible growth defects, root growth in these lines was determined in comparison to *mus81-1* and wild type plants (Fig 4). Root length in *mus81-1* mutants was significantly reduced compared to that of wild type plants. Interestingly, both the *top3A-2 mus81-1* complementation line lacking all C-terminal zinc-fingers and the line lacking only zinc-finger T1, exhibit a significantly reduced root length compared to *mus81-1*, while this was not the case for the other lines. Thus, we were able to show that the zinc-finger domain T1 seems to be required for TOP3α function in the repair of replication-associated DNA damage in a pathway parallel to MUS81.

### Toxic recombination intermediates can be removed by a HJ resolvase

**Fig 5:**
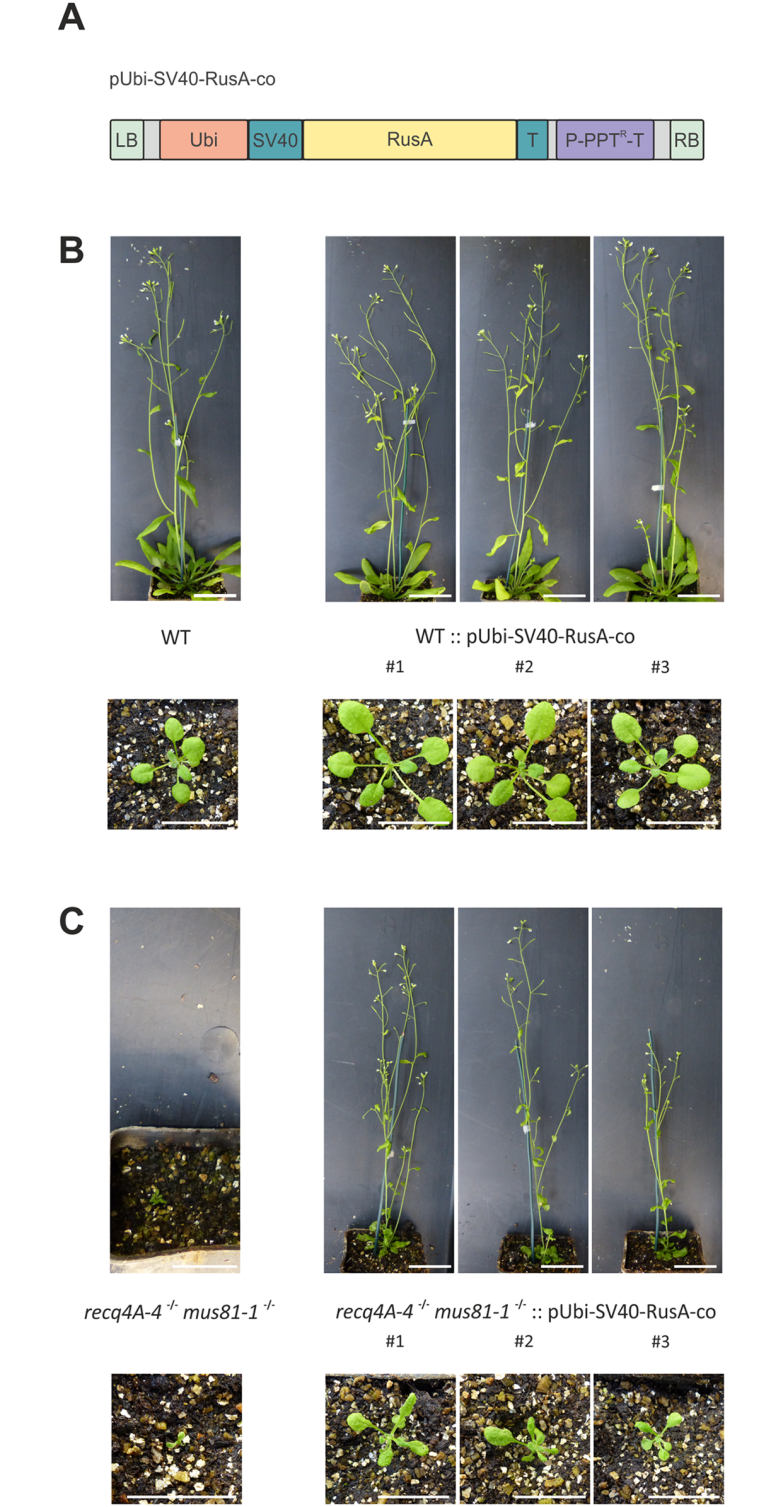
Rescue of growth defects by heterologous expression of RusA. (A) Schematic illustration of the pUbi-SV40-RusA-co construct. The construct is flanked by the two border sequences (left border, LB; right border RB) for T-DNA integration. For *Arabidopsis thaliana* codon optimized RusA was equipped with a SV40 nuclear localization signal at the N-terminus. For expression of the RusA construct, a Ubiquitin4-2 (Ubi) promotor from *Petroselinum crispum* and 35S terminator (T) from cauliflower mosaic virus were used. A phosphinotricin (PPT) resistance cassette with promotor (P) and terminator (T) served for plant selection. (B) Heterologous expression of *RusA* in wild type (WT) plants. Three-week-old plantlets and six-week-old plants of three independent WT lines harbouring the RusA construct are compared to WT plants. The WT lines expressing the RusA construct do not display any differences in growth compared to WT plants. Scale bar = 2 cm. (C) Heterologous expression of *RusA* in *recq4A-4 mus81-1* mutants. Three-week-old plantlets and six-week-old plants of three independent *recq4A-4 mus81-1* lines containing the RusA construct are compared to the original *recq4A-4 mus81-1* mutant. While *recq4A-4 mus81-1* mutants depict a synthetic lethal phenotype, where plants are able to germinate but die after the formation of several deformed leaves, the expression of *RusA* in this line leads to viable plants. All *recq4A-4 mus81-1* lines expressing *RusA* show a viable and fertile, but growth restricted phenotype. Scale bar = 2 cm.

We wondered about the specific nature of the DNA repair intermediates in which the zinc-finger domain T1 of TOP3α is specifically required for. The repair of DSBs via HR, results in the formation of recombination intermediates such as the dHJ. The resolution of this intermediate can be performed by endonucleolytic activity of HJ-resolvases or by RTR-complex-mediated dissolution [59,60]. The simultaneous elimination of both repair pathways was shown to lead to persisting toxic recombination intermediates, ultimately resulting in lethality. We found for *A*. *thaliana*, that the simultaneous defect of the resolvase MUS81 and the RTR-complex partner RECQ4A leads to synthetic lethality in the double mutant [46]. Prokaryotes harbour distinct structure specific resolvases like the *E*. *coli* RusA resolvase coded by a gene of the defective prophage DLP12. The overexpression of *RusA* in human cells defective in the HJ resolvase SLX4 and the RTR-complex partner BLM, was able to rescue lethality, thereby demonstrating dHJs as the cause for the accumulation of chromosomal damage [61]. To test whether such an approach is transferable to Arabidopsis, we cloned a construct consisting of the *E*. *coli RusA* open reading frame, codon-optimized for Arabidopsis, with a SV40 nuclear localization signal attached to the 5’ end, under the control of the constitutive active Ubiquitin4-2 promotor from *Petroselinum crispum* and the 35S cauliflower mosaic virus terminator (Fig 5A). The construct was stably integrated by agrobacterium-mediated transformation into wild type plants and *mus81-1* -/- *recq4A-4* +/- double mutants. Three independent, homozygous lines of each were established. While wild type plants harbouring the construct were not affected in their growth, expression of *RusA* in *mus81-1 recq4A-4* double mutants resulted in a rescue of the synthetic lethal phenotype (Fig 5B and 5C). As previously shown, *mus81-1 recq4A-4* double mutants are able to germinate but die after the formation of one to two leaves, but the expression of *RusA* in this line leads to viable plants that reach seed ripening. This result demonstrated that we can use heterologous expression of *RusA* to resolve persisting HJ structures in somatic plant cells.

**Fig 6:**
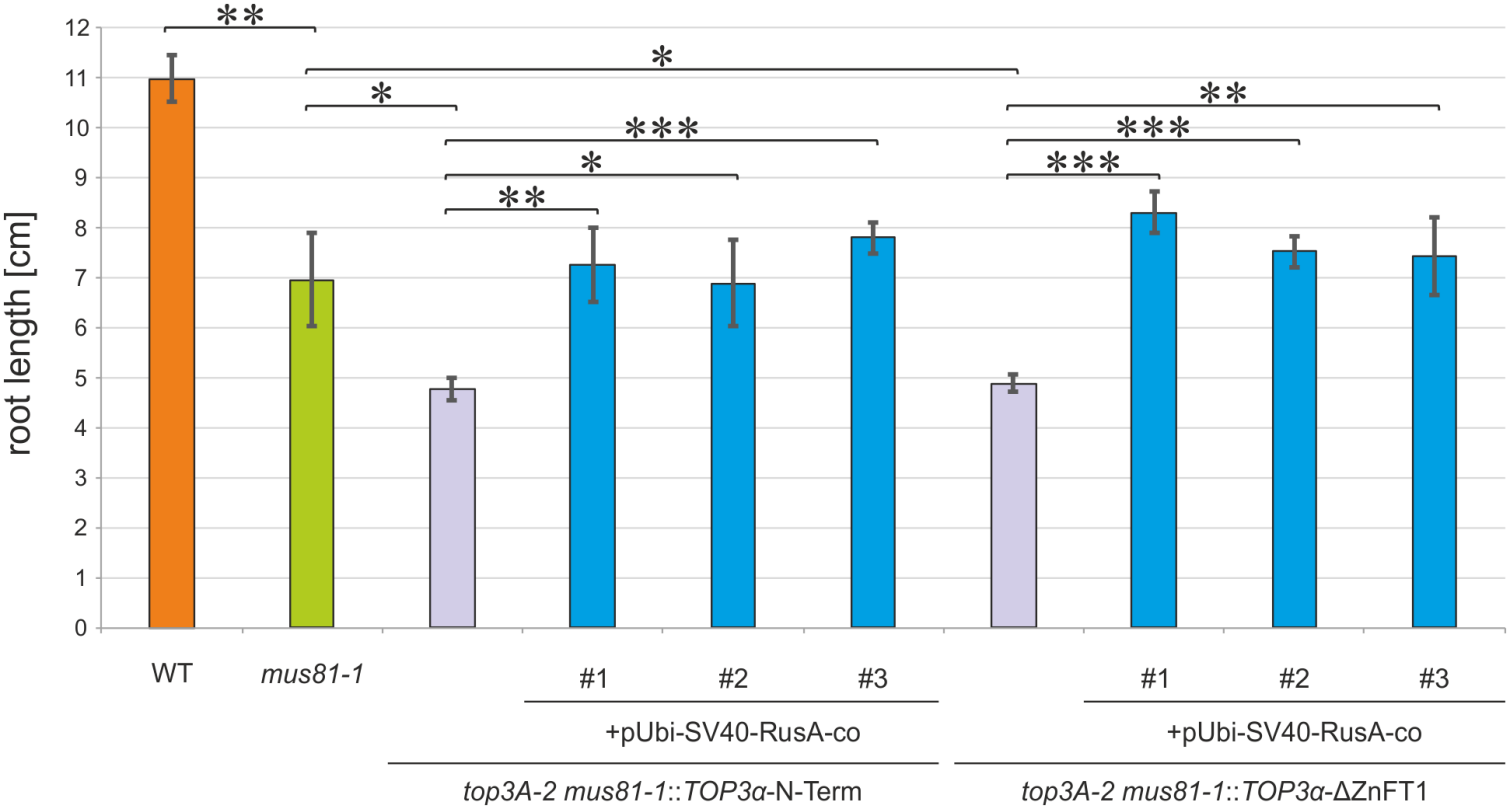
Rescue of reduced root length in *top3A-2 mus81-1* complementation lines lacking zinc-finger T1 by expression of *RusA*. Root length of ten-day-old plants from each three *top3A-2 mus81-1*::*TOP3α*-N-Term/ΔZnFT1 lines independently harbouring the RusA rescue construct was determined in comparison to the original *top3A-2 mus81-1* complementation lines, the *mus81-1* mutant and wild type (WT) plants. Four independent assays were performed and mean values with standard deviation (error bars) were calculated. In the *mus81-1* mutant, a significantly reduced root length compared to the WT could be observed. For *top3A-2 mus81-1*::*TOP3α*-N-Term/ΔZnFT1 lines a further statistically significant reduction of root length was shown. The additional expression of *RusA* in these lines restores root length to the same extent as in the *mus81-1* mutant in all analysed lines. Statistical differences were calculated using a two-tailed t-test with unequal variances: * p < 0.05, ** p < 0.01, *** p < 0.001.

We speculated that the cause for the reduced root length in *top3A-2* complementation lines lacking the zinc-finger domain T1 in the absence of MUS81, were due to persisting dHJ-like recombination intermediates. To test this hypothesis, we integrated the *RusA* rescue construct into these lines by agrobacterium-mediated transformation and established three independent lines for each. For the quantification of this rescue approach, we determined root growth in the original *top3A-2 mus81-1*::*TOP3α*-N-Term and *top3A-2 mus81-1*::*TOP3α*-ΔZnFT1 lines in comparison to three independent lines each harbouring the *RusA* construct, *mus81-1* mutants and wild type plants (Fig 6). As shown before, compared to wild type plants, *mus81-1* mutants exhibit a reduced root length that is further diminished by the deletion of all TOP3α zinc-finger domains or the single deletion of TOP3α zinc-finger T1 in the respective transformants. The additional expression of RusA in these lines could now rescue this phenotype so that root length was undistinguishable from *mus81-1* mutants. This indicates that unresolved HJ-like structures accumulate in lines lacking both *mus81-1* and the TOP3α zinc-finger domain T1. Thus, the domain seems to be required for targeting the topoisomerase to these specific structures, most probably by direct DNA binding or by interaction with another protein.

## Discussion

Topoisomerase 3α is the crucial partner of the RTR-complex by catalysing the decatenation of recombination intermediates [12]. Because of this essential function, *in vivo* analyses of TOP3α homologs are difficult in most eukaryotes. Here, we were able to demonstrate that the null mutant of TOP3α is viable in the plant model organism, *Arabidopsis thaliana*. This enabled us to perform *in vivo* analyses of TOP3α domain functions by complementation studies, revealing an important role for the conserved zinc-finger domain T1 in the processing of Holliday junction like DNA repair intermediates. We assume that this function is conserved amongst eukaryotes.

Different mutant lines for *top3α* in Arabidopsis have been characterized so far, exhibiting a wide variation of phenotypes from only mild meiotic abnormalities over strong growth defects associated with meiotic catastrophe to lethality [27,29,31,34]. Due to its strongest phenotype and as lethality was also observed in mutants from other eukaryotes, until now the *top3A-1* mutant was regarded as complete knock-out. By CRISPR/Cas induced mutagenesis, we could now demonstrate that indeed the viable T-DNA insertion line *top3A-2* mutant line shows the true null phenotype, as four different, newly generated mutant lines were indistinguishable to *top3A-2*. Nevertheless, we still questioned as to how the lethal *top3A-1* phenotype could be explained. We therefore sequenced the complete gene in this mutant, as only the T-DNA insertion sites were determined before. The analysis revealed an additional deletion 5’ of the T-DNA (S11 Fig). This deletion leads to a premature stop codon, resulting in a truncated TOP3α protein not only lacking all C-terminal zinc-fingers, but also part of the central domain. Interestingly, this mimicks our complementation approach with *TOP3α*-Central (leading to a catalytically inactive TOP3α lacking the C-terminal zinc-finger domains), in both genotype and phenotype. Thus, we could solve the puzzle of the *top3α* mutant phenotype *in Arabidopsis*: the viable but sterile *top3A-2* with its insertion in the central domain is representing the null-phenotype, as well as the four new CRISPR/Cas mutants with frameshifts in the TOPRIM domain. Intriguingly, these two domains are essential for catalytic activity of the protein [36,54,55,62,63]. Mammalian *top3α* mutants are embryo lethal and for *C*. *elegans* and *D*. *melanogaster* essential functions were also described for TOP3α [18–20]. For fungal TOP3α homologs, mutant phenotypes differ as *top3* mutants in *S*. *pombe* are lethal whilst they are viable with a wide number of defects in *S*. *cerevisiae* [64,65]. Interestingly, *top3α* mutants from Arabidopsis seem to resemble mutants from baker’s yeast, differing from mammalian mutants. The detailed characterization of the new At*top3α* mutant lines enabled us to confirm all currently described *top3A-2* mutant characteristics, including the abortion of meiosis [31]. Furthermore, a reduced root length, based on severe cell death in the root meristem, could be observed, hinting to a role of TOP3α in the repair of replication-associated DNA damage. This is in accordance with previous results demonstrating a mitotic defect in *top3A-2* mutants that might originate from defects in DNA repair [31]. Additionally, a role for TOP3α in the repair of interstrand CLs was elucidated for the first time. As we were recently able to demonstrate a role for the RTR helicase RECQ4A in interstrand CL repair, we assume that the RTR-complex as a whole is involved in interstrand CL repair in Arabidopsis [66].

The demonstration of a viable *top3α* mutant phenotype for Arabidopsis laid the ground for our extensive *in vivo* analyses of domain functions via complementation studies. Due to the lack of a functional TOP3α antibody, we were not able to quantify the expression of the different TOP3α variants in our complementation lines, directly. As a result, we established at least three independent transgenic lines for each approach and reproduced the complementation experiments with *TOP3α*, *TOP3α*-ΔTOPRIM, *TOP3α*-Y342F, *TOP3α*-N-Term and *TOP3α*-Central, using a second independent *top3α*-mutant line. We are convinced that by supplying these different controls, all our conclusions regarding our complementation analyses are justified even without direct protein quantification. The complementation of all mutant phenotypes by the expression of the full-length *TOP3α* gene in *top3A-2* and *top3A-6* confirmed both the general experimental approach and the usability of the mutant lines. As wild type lines containing the different complementation constructs exhibit a phenotype indistinguishable from wild type plants, a negative effect in the presence of intact TOP3α could be excluded, ruling out possible artefacts.

Both the N-terminal TOPRIM domain and the catalytic tyrosine of TOP3α are essential for topoisomerase activity. Tyrosine substitution with a phenylalanine abolishes enzymatic activity in human and yeast TOP3α homologs [54,67]. Furthermore, for single amino acids within the TOPRIM domain of *E*. *coli* Top1, functions in DNA religation and transesterification were demonstrated [62,63]. Indeed, both the TOPRIM deletion and the substitution of the catalytic tyrosine lead to identical phenotypes in Arabidopsis, but the phenotype was surprisingly much stronger than with the null mutants: expression of either complementation construct in *top3α* mutants lead to an aggravated, embryo lethal phenotype. This effect could only be observed in homozygous *top3α* mutants, so that we are able to conclude that the expression of an AtTOP3α variant without catalytic activity can induce lethality in the absence of functional TOP3α protein. Surprisingly, the combined deletion of the TOPRIM domain and the C-terminal zinc-finger domains in a construct, enabled transformed *top3α* mutants to retrieve growth to a certain extent. Thus, the presence of the C-terminal zinc-finger domains (ZFDs) is essential for inducing lethality by the non-functional topoisomerase in the *top3α* mutants. However, this leads to the question as to why does the presence of the ZFDs trigger tethality? The easiest explanation is that the ZFDs are indeed targeting the aberrant protein to DNA repair intermediates, which due to masking, cannot be resolved anymore by other enzymes, leading to cell death. This binding could be achieved either indirectly by the interaction with other proteins, or directly to specific DNA structures. We strongly favour the second hypothesis as a role in the binding of ssDNA was demonstrated for the C-terminal part of *E*. *coli* Top1 [68]. Recent crystal structure analyses further confirmed ssDNA binding by C-terminal ZFDs [69]. Indication of a direct interaction of the zinc-finger domains in TOP3α homologs in animals comes from *in vitro* analyses of *D*. *melanogaster* TOP3α, showing ssDNA binding activity [70]. To address this question biochemically, we expressed a cDNA containing the ZFDs of AtTOP3α in *E coli* and insect cells. Unfortunately, the obtained proteins aggregated, which excluded the performance of DNA binding assays. Therefore, we had to use a genetic approach to further characterize what kind of DNA molecule the ZFDs might be able to interact with. Transformation of a TOP3α construct lacking only the ZFDs into the *top3A-2* mutant restored all reported phenotypes, including differences in the transcriptome. Thus, we came to the conclusion that the C-terminal ZFDs in AtTOP3α do not contribute essentially to the primary functions of the protein, but are only required by AtTOP3α for the processing of specific DNA structures, which we set out to define in more detail.

As TOP3α acts as a partner of the conserved RTR complex in the dissolution of homologous recombination intermediates like dHJs, we speculated that the ZFDs might be required for targeting the topoisomerase to this class of intermediates via DNA binding. In the case of a lack of the topoisomerase, unresolved recombination intermediates have to be processed by other enzymatic activities to safeguard the survival of the cell. There is strong evidence from various eukaryotes that the processing of these HJ-like recombination intermediates is mediated by the resolvase MUS81. MUS81 forms a conserved complex together with its partner EME1, that catalyses the resolution of intermediates like dHJs, D-loops and blocked replication forks, leading to both CO and NCO products [39,41,71]. A severe growth retardation phenotype was already described for the *recq4A-4 mus81-1* double mutant, demonstrating the parallel involvement of both pathways [46]. As we were able to abolish the growth defect in this double mutant by knocking out the homologous recombination pathway, we could demonstrate that the structures are indeed HR intermediates [47]. The embryo lethal phenotype of *top3A-2 mus81-1* double mutants, which we detected in the current study, highlights the importance of TOP3α in the processing of these intermediates in the absence of MUS81. By introduction of full-length TOP3α, but also TOP3α variants lacking zinc-finger domains GRF or CCHC1/2, we were able to restore growth defects in the double mutant to *mus81-1* single mutant level, indicating no special role of these three zinc-finger domains in the resolution of recombination intermediates in the absence of MUS81. However, this was not the case for variants harbouring a deletion of all zinc-finger domains nor the sole deletion of zinc-finger T1, as complementation lines exhibited a reduced root length compared to *mus81-1* single mutants. Our results therefore indicate a unique role of the TOP3α zinc-finger T1, which is conserved in various eukaryotes, in targeting the HR intermediates.

As classical HR intermediates are single or double HJs, we thought these would be the most likely structure for TOP3α zinc-finger T1 in targeting. Thus, HJs should prevail in the *top3A-2 mus81-1* double mutants complemented with a topoisomerase that lacks zinc-finger T1. If these structures are indeed HJs, we assumed that we should be able to counteract their accumulation and reverse the root growth defect by the expression of a HJ resolvase. The bacterial RusA is coded by a gene of the defective prophage DLP12 and is able to process structural specific HJs [72,73]. Confirming our hypothesis, we were able to show that the expression of *RusA* in *recq4A-4 mus81-1* double mutants was able to rescue viability, confirming the accumulation of HJ-like structures in the absence of both pathways. Most importantly, we were also able to demonstrate that *RusA* expression can rescue the growth defects caused by the deletion of TOP3α zinc-finger domain T1 in the absence of MUS81. This clearly demonstrates that TOP3α does not process specifically HJ-like structures efficiently in the absence of zinc-finger T1. As root length was only restored to *mus81-1* mutant level, there seems to be other persisting repair intermediates, that cannot be resolved by RusA. These intermediates must require the function of the endonuclease MUS81, that is able to process various other DNA structures besides HJs, such as flaps [43].

We suggest that the zinc-finger domain T1 from AtTOP3α targets the topoisomerase to HJ-like structures by directly mediating structure specific binding, a prerequisite for the resolution of these structures. It has recently been suggested that the C-terminal domain of AtTOP3α is required for a sub-specialization of meiotic CO-control [34]. As we now have strong indications from somatic cells, that the zinc-finger domain T1 from AtTOP3α is involved in targeting the enzyme to specific HJ-like intermediates formed by HR, it is highly likely that the same function is required for CO-control in meiosis as well.

## Materials and methods

### Plant material and growth conditions

All used *Arabidopsis thaliana* lines were in the Columbia (Col-0) background. The T-DNA insertion lines *top3A-1* (SALK_139357), *top3A-2* (GABI_476A12), *mus81-1* (GABI_113F11) and *recq4A-4* (GABI_203C07) from the SALK and GABI-Kat collections were previously described [29,31,46]. For the further characterization of TOP3α in Arabidopsis, the additional mutant lines *top3A-3*, *top3A-4*, *top3A-5* and *top3A-6* were generated by Cas9-mediated mutagenesis of Arabidopsis wild type plants, as previously described [51]. The genotypes of all mutant lines were verified by PCR. To confirm the mutant background during the establishment of transformed *top3A-2* and *top3A-6* lines, individual primer combinations were used to avoid primer binding in the complementation construct (Supporting Information).

Plants grown in the greenhouse were cultivated in a 1:1 mixture of Floraton 3 (Floragard, Oldenburg, Germany) and vermiculite (2-3mm, Deutsche Vermiculite Dämmstoff, Sprockhövel, Germany) with 16 h light and 8 h darkness at 22 °C. For assays, plants were cultivated in axenic culture, as described before [28].

### Plasmid construction and plant transformation

As a basis for the construction of complementation vectors containing different *TOP3α* variants, the binary plasmid 35SpBARN was used [74]. The 35S promotor and nopalin synthase terminator were substituted with a multiple cloning site, thus resulting in the plasmid pBARN-MCS. All *TOP3α* constructs were cloned from Arabidopsis gDNA by In-Fusion cloning (Clontech) and the sequence 1173 bp upstream of the At*TOP3α*-5’UTR served as promotor, while the sequence 56 bp downstream of the At*TOP3α*-3’UTR served as terminator. The sequences and primer combinations for the generation of the different fragments, with suitable overhangs for cloning, is given in the Supporting Information.

The construct for the overexpression of *RusA* in Arabidopsis was based on the binary plasmid pPZP201, containing a phosphinothricin (PPT) resistance cassette under the control of the CaMV 35S gene promoter and terminator [30,75]. For the expression of *RusA*, the Ubiquitin4-2 promotor from *Petroselinum crispum* was inserted into the vector. The coding sequence of *RusA* was synthesised by Gene Art (Thermo Fisher Scientific) and codon optimized for *Arabidopsis thaliana*. Furthermore, an *Arabidopsis thaliana* codon optimized nuclear localization sequence based on the Simian-virus 40 T-antigen was added to the 5’end of RusA, with a 3’ start codon. As terminator sequence, the CaMV 35S gene terminator was used. Restriction cutting sites for BSU36I and NCOI were added to the synthesized product, which was integrated into the vector by restriction digestion followed by ligation. For the transformation of the overexpression construct into TOP3α complementation lines, the PPT resistance cassette was exchanged with a gentamycin resistance. Sequences of all vectors were verified by Sanger sequencing.

Transformation of plants was performed using *Agrobacterium tumefaciens* with the floral dip method [76]. Primary transformants were identified using the resistance cassette contained within the T-DNA. In the T2 generation, single locus lines were identified by a 3:1 Mendelian segregation of the resistance cassette. In the T3 generation, lines containing the transgene homozygously were identified and seeds propagated from these lines were used for further experiments.

### Sensitivity assays

Sensitivity assays were performed as previously described [29]. Seeds were surface sterilised and sown on GM medium. After incubation for two weeks, five plantlets were transferred into a well of a six-well plate containing 5 ml liquid GM medium for the untreated control or 4 ml for genotoxin induced approaches. 1 ml of the respective genotoxin solution was added the following day to obtain the final genotoxin concentration. After an additional 13 days of incubation, the fresh weight of the plants was measured. For analyses, the fresh weight of treated plants was calculated in relation to the fresh weight of the untreated control.

### HR assays

HR assays were performed as described using the IC9 reporter construct [29,53]. Seeds were surface sterilised and sown on GM medium. After incubation for two weeks, 30 plantlets were transferred into halved Petri dishes containing 10 ml liquid GM medium and incubated for an additional week. Then the histochemical GUS staining was performed as described. For quantifying the HR rate, blue sectors of each plant were counted using a binocular microscope.

### Root length assay

To analyse root length, surface sterilised seeds were sown on square plates with GM medium containing 1% plant agar. After 10 days of vertical incubation, the plates were photographed on a dark background. Analysis of root length was performed using ImageJ plugin SmartRoot [77]. The analysis of cell death in the root meristem by propidium iodide staining was performed as previously described [78].

### Embryo analysis

To analyse embryo development in Arabidopsis seeds, immature siliques were harvested and directly opened with a cannula. The freed seeds were then transferred onto a microscope slide with 80 μl clearing solution (2 g chloral hydrate, 250 μl glycerol, 500 μl ddH_2_O) and covered with a coverslip. After incubation for at least 1 h at room temperature, the slides were analysed by light microscopy using a DIC filter.

### Fertility analysis

For the fertility analysis of Arabidopsis lines, plants were grown in the greenhouse. Five mature siliques of at least five plants per line were transferred into 70% EtOH and incubated overnight. The silique length and seeds per silique were determined with a binocular microscope.

### DAPI staining of male meiocytes

Chromatin preparation of male meiocytes was performed as previously described [79].

### RNA-seq

For transcriptome analyses, total RNA from two-week-old plantlets was extracted using the RNeasy Plant Mini Kit (Qiagen GmbH, Hilden, Germany). Three biological replicates were performed. Library preparation and sequencing was performed by Eurofins Genomics (Ebersberg, Germany). Sequencing was executed on an Illumina HiSeq with a sequencing mode of 1x100 bp reads. For differential gene expression analysis, reads were aligned to the Arabidopsis reference genome (TAIR10) and the CLC Genomics Workbench 7.5 was used with default parameters. Genes showing an FDR < 0.05 were considered as significantly differentially expressed.

### Accession numbers

Sequence data from this article can be found with the following Arabidopsis AGI locus identifiers, AtTOP3α: At5g63920, AtMUS81: At4g30870, AtRECQ4A: At1g10930. The RNA-seq data has been deposited in NCBI’s Gene Expression Omnibus [80] and is accessible through the GEO Series accession number GSE116582 (https://www.ncbi.nlm.nih.gov/geo/query/acc.cgi?acc=GSE116582).

## Supporting information

S1 Fig Phenotype of At*top3α* T-DNA mutants. (A) Six-week-old *top3A-2* mutant plants (left) exhibit fasciated organs and a dwarf phenotype. Two-week-old mutant plantlets already show growth defects like deformed leaves. (B) Two-week-old *top3A-1* mutant plants feature deformed cotyledons and no roots are formed.
(PDF)

S2 Fig cDNA analysis in At*top3α* mutant lines. cDNA sequences of CRISPR/Cas9 induced *top3α* mutant lines were aligned with wild type (WT) sequences. The start codon is depicted in blue, sequences differing from the WT in red. All mutations from the different mutant lines lead to a frameshift, generating a premature stop codon (red box).
(PDF)

S3 Fig Recombination rate and genotoxin sensitivity of At*top3α* mutant lines. (A) The number of blue sectors per plant in *top3A-2*, *top3A-4* and *top3A-6* in comparison to the wild type (WT) is depicted. Recombination rate was determined using the IC9C reporter construct. All three mutant lines exhibit an elevated recombination rate compared to the WT. (B) Relative fresh weight of *top3A-2*, *top3A-4* and *top3A-6* mutant lines and WT plants in response to cisplatin, methylmethanesulfonate (MMS), camptothecin (CPT) and mitomycin C (MMC) was determined. All mutant lines show a reduced fresh weight in comparison to the WT after treatment with the respective genotoxins. Significant differences to the WT control were calculated using a two-tailed t-test with unequal variances: * p < 0.05, ** p < 0.01, *** p < 0.001.
(PDF)

S4 Fig Fertility analysis in At*top3α* mutant lines. Average silique length (A) and seeds per silique (B) were determined for *top3A-2*, *top3A-3*, *top3A-4*, *top3A-5* and *top3A-6* mutant lines in comparison to the wild type (WT). All *top3α* mutant lines exhibited a reduced silique length in comparison to the WT and no seeds were observed. Significant differences to the WT control were calculated using a two-tailed t-test with unequal variances: * p < 0.05, ** p < 0.01, *** p < 0.001. (C) Detailed analysis of meiosis in *top3A-2*, *top3A-4* and *top3A-6* mutant lines compared to that of WT. The complete course of meiosis was observed in pollen mother cells of WT plants, while *top3α* mutants show defects such as fragmentation and no stages from meiosis II could be observed.
(PDF)

S5 Fig Growth phenotype of wild type plants containing *TOP3α* complementation constructs. Six-week-old wild type (WT) plants containing the different *TOP3α* complementation constructs *TOP3α*-ΔTOPRIM, *TOP3α*-Y342F, *TOP3α*-Central (A) and *TOP3α*-N-Term, *TOP3α*-ΔZnFT1, *TOP3α*-ΔZnFCCHC1, *TOP3α*-ΔZnFGRF, *TOP3α*-ΔZnFCCHC2 (B) are shown in comparison to the WT. All complementation lines exhibit a growth phenotype indistinguishable from the WT.
(PDF)

S6 Fig Complementation of *top3A-2* mutants with full-length *TOP3α*. Root length (A), recombination rate (B), genotoxin sensitivity (C) and fertility (D) of three independent *top3A-2*::*TOP3α* lines was determined in comparison to the *top3A-2* mutant and wild type (WT) plants. The complementation lines exhibited a complete reversal of all mutant phenotypes. Significant differences to the WT control were calculated using a two-tailed t-test with unequal variances: * p < 0.05, ** p < 0.01, *** p < 0.001.
(PDF)

S7 Fig Complementation of *top3A-6* mutants with different TOP3α variants. (A) Two-week-old seedlings and five-week-old plants from three individual *top3A-6*::*TOP3α* complementation lines compared to *top3A-6* mutants and wild type (WT) plants. The characteristic growth defects of *top3A-6* could be fully complemented by expression of *TOP3α* in all three complementation lines, leading to a growth phenotype indistinguishable to WT plants. (B) Two-week-old plantlets of three individual *top3A-6*::*TOP3α*-Central complementation lines compared to *top3A-6* mutants and WT plants. While *top3A-6* mutant lines exhibit characteristic growth defects with dark and deformed leaves, expression of *TOP3α*-Central in this line leads to an enhanced growth defect. Plants feature only the cotyledons that are deformed and no roots are formed. (C) Two-week-old plantlets and five-week-old plants of four individual *top3A-6*::*TOP3α*-N-Term complementation lines are compared to *top3A-6* mutants and WT plants. The growth defects of *top3A-6* mutant plants could be complemented completely by expression of *TOP3α*-N-Term leading to plants indistinguishable from the WT.
(PDF)

S8 Fig Complementation of *top3A-2* mutants with *TOP3α*-N-Term. Root length (A), recombination rate (B), genotoxin sensitivity (C) and fertility (D) of four independent *top3A-2*::*TOP3α*-N-Term lines was determined in comparison to the *top3A-2* mutant and wild type (WT) plants. The complementation lines exhibited a complete reversal of all mutant phenotypes. Significant differences to the WT control were calculated using a two-tailed t-test with unequal variances: * p < 0.05, ** p < 0.01, *** p < 0.001.
(PDF)

S9 Fig Expression analysis of *HEI10* and *MRD1* in *top3A-2*::*TOP3α*-N-Term. The expression of *HEI10* and *MRD1* in *top3A-2*, *top3A-2*::*TOP3α*-N-Term and wild type (WT) plants was tested by qRT-PCR analysis with two primer pairs each. Three independent assays were performed and mean values with standard deviation (error bars) were calculated. Statistical differences were calculated using a two-tailed t-test with unequal variances: * p < 0.05, ns = not significant. (A) For *HEI10*, the expression at the 5’ and 3’ end of the gene was comparable in all analysed lines. (B) For *MRD1*, the expression at the 5’ end of the gene was significantly increased in *top3A-2*::*TOP3α*-N-Term (69x) compared to the WT.
(PDF)

S10 Fig Embryo development in *top3A-2* +/- *mus81-1* double mutants. Depicted are representative embryos of exemplary *top3A-2* +/- *mus81-1* double mutants compared to wild type (WT), *top3A-2* +/- and *mus81-1* embryos. All lines showed complete embryo development leading to mature embryos. In heterozygous *top3A-2 mus81-1* double mutant lines, a Χ^2^ test confirmed a ratio of ¼ seeds with lacking or deformed embryos, corresponding to the amount of homozygous *top3A-2 mus81-1* double mutants.
(PDF)

S11 Fig gDNA analysis of *TOP3α* exon 15 in *top3A-1*. Depicted is an alignment of the gDNA sequence from *TOP3α* exon 15 of *top3A-1* and the wild type (WT) sequence. Sequences differing from the WT are depicted in red. In *top3A-1*, a 44 bp deletion was identified on mRNA level leading to a premature stop codon in frame (red box). On protein level, this results in a truncated TOP3α protein, missing all C-terminal zinc-finger domains and the last 21 amino acids of the central domain.
(PDF)

S1 Table Statistical analysis of embryo development in *top3A-6* +/- ::*TOP3α*-Y342F/ΔTOPRIM. (PDF)

S2 Table Statistical analysis of embryo development in *top3A-2* +/- ::*TOP3α*-Y342F/ΔTOPRIM. (PDF)

S3 Table Differentially expressed genes in *top3A-2* and *top3A-2*::*TOP3α*-N-Term. (PDF)

S4 Table Statistical analysis of embryo development in *top3A-2* +/- *mus81-1*. (PDF)

S5 Table Primer combinations for genotyping. (PDF)

S6 Table Primer sequences for genotyping. (PDF)

S7 Table Primer combinations for In-Fusion cloning fragment amplification. (PDF)

S8 Table Primer sequences for In-Fusion cloning. (PDF)

S1 Method qRT-PCR analysis of *HEI10* and *MRD1* gene expression. (PDF)

S2 Method 5’ RACE analysis of *HEI10* cDNA. (PDF)
