# Supplementary figures and images for: The topoisomerase 3α zinc-finger domain T1 of Arabidopsis thaliana is required for targeting the enzyme activity to Holliday junction-like DNA repair intermediates
Source: PLoS Genet. 2018 Sep 17;14(9):e1007674. doi: 10.1371/journal.pgen.1007674 (PMC6160208; doi:10.1371/journal.pgen.1007674)

**A**

*top3A-2*

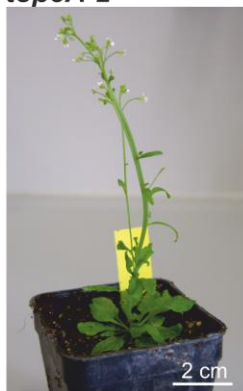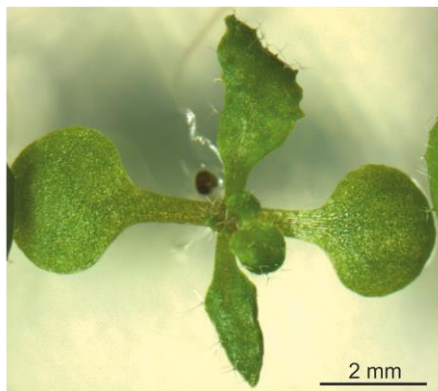

**B**

*top3A-1*

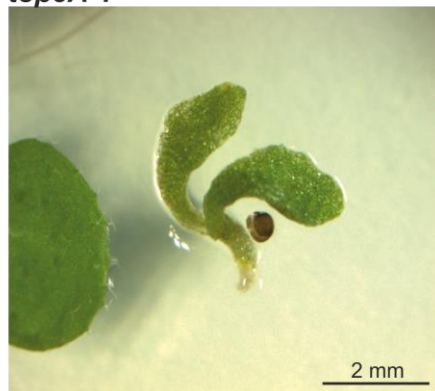

Supplement: S1 Fig — (A) Six-week-old top3A-2 mutant plants (left) exhibit fasciated organs and a dwarf phenotype. Two-week-old mutant plantlets already show growth defects like deformed leaves. (B) Two-week-old top3A-1 mutant plants feature deformed cotyledons and no roots are formed. (PDF) [file pgen.1007674.s001.pdf]

**A**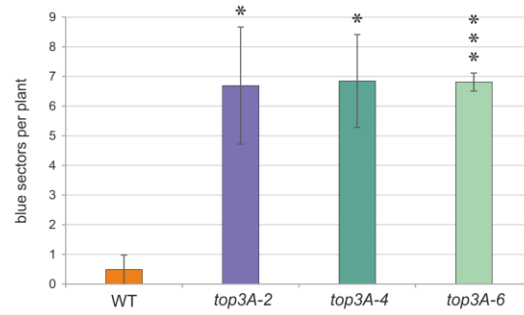**B**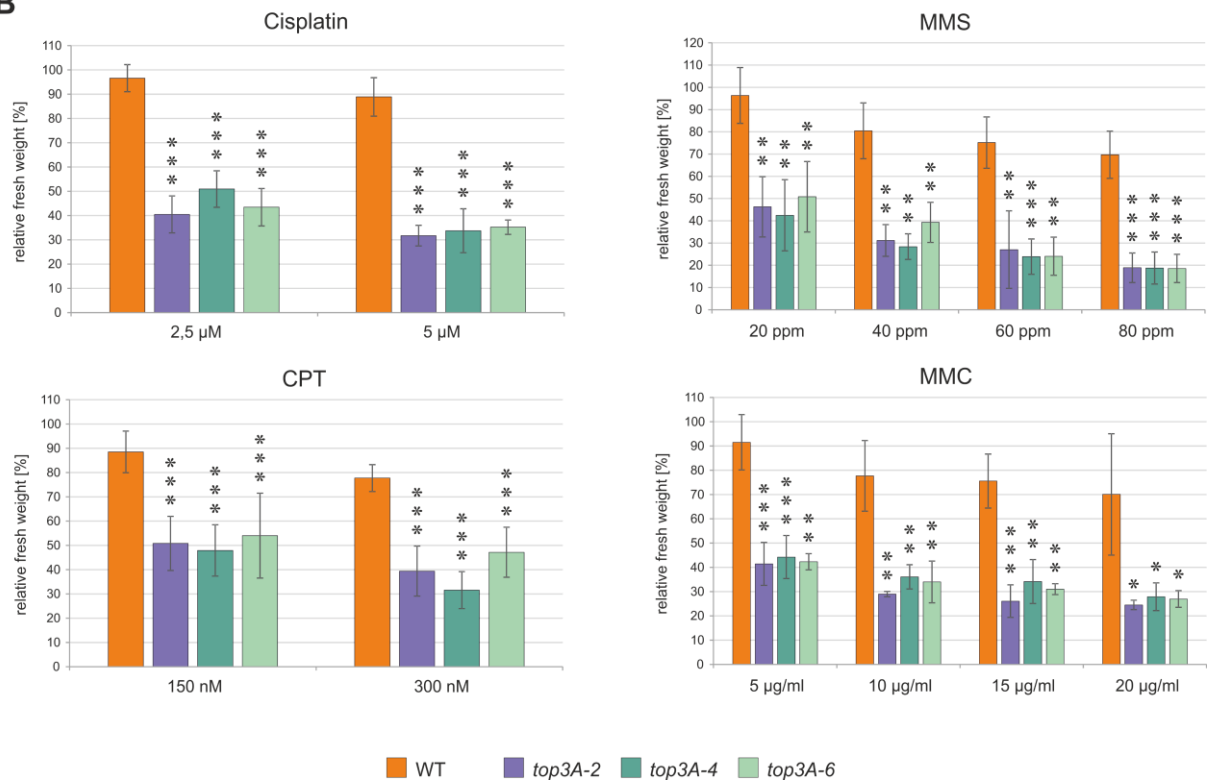

Supplement: S3 Fig — (A) The number of blue sectors per plant in top3A-2, top3A-4 and top3A-6 in comparison to the wild type (WT) is depicted. Recombination rate was determined using the IC9C reporter construct. All three mutant lines exhibit an elevated recombination rate compared to the WT. (B) Relative fresh weight of top3A-2, top3A-4 and top3A-6 mutant lines and WT plants in response to cisplatin, methylmethanesulfonate (MMS), camptothecin (CPT) and mitomycin C (MMC) was determined. All mutant lines show a reduced fresh weight in comparison to the WT after treatment with the respective genotoxins. Significant differences to the WT control were calculated using a two-tailed t-test with unequal variances: * p < 0.05, ** p < 0.01, *** p < 0.001. (PDF) [file pgen.1007674.s003.pdf]

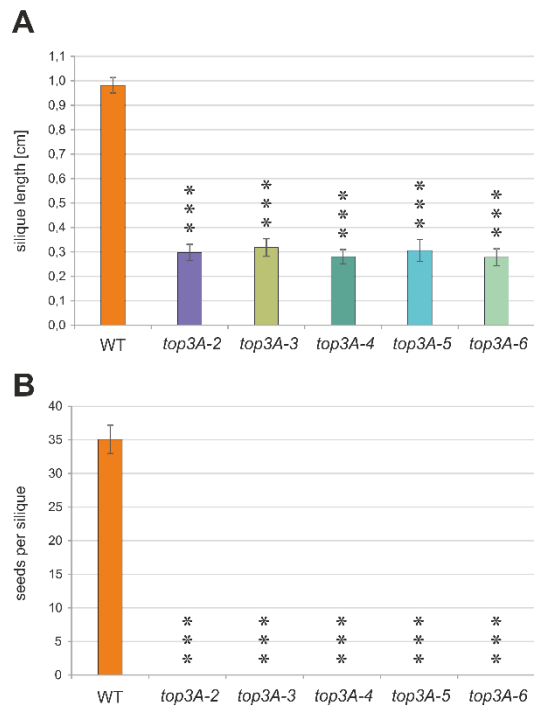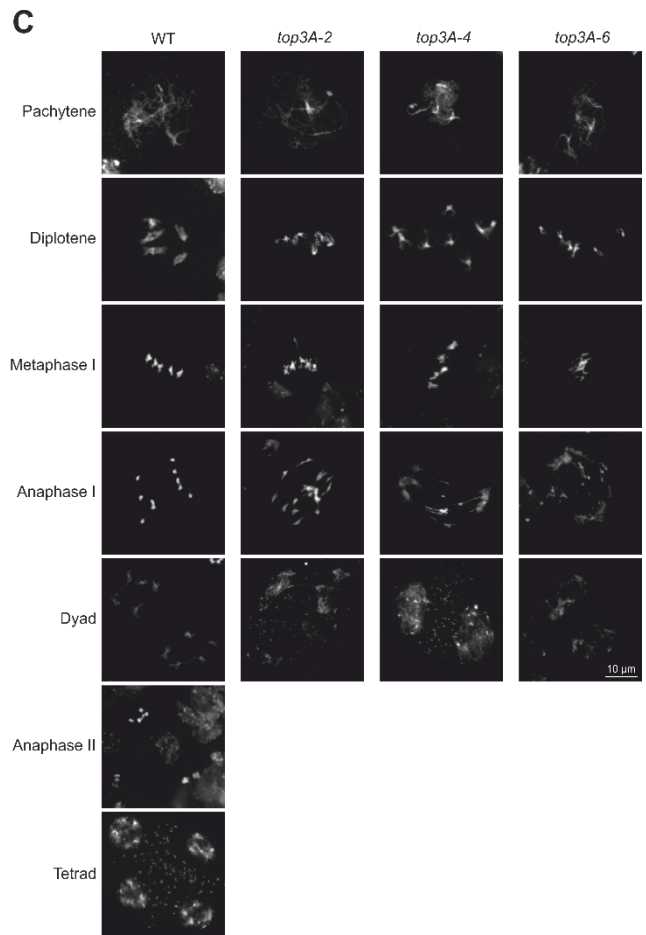

Supplement: S4 Fig — Average silique length (A) and seeds per silique (B) were determined for top3A-2, top3A-3, top3A-4, top3A-5 and top3A-6 mutant lines in comparison to the wild type (WT). All top3α mutant lines exhibited a reduced silique length in comparison to the WT and no seeds were observed. Significant differences to the WT control were calculated using a two-tailed t-test with unequal variances: * p < 0.05, ** p < 0.01, *** p < 0.001. (C) Detailed analysis of meiosis in top3A-2, top3A-4 and top3A-6 mutant lines compared to that of WT. The complete course of meiosis was observed in pollen mother cells of WT plants, while top3α mutants show defects such as fragmentation and no stages from meiosis II could be observed. (PDF) [file pgen.1007674.s004.pdf]

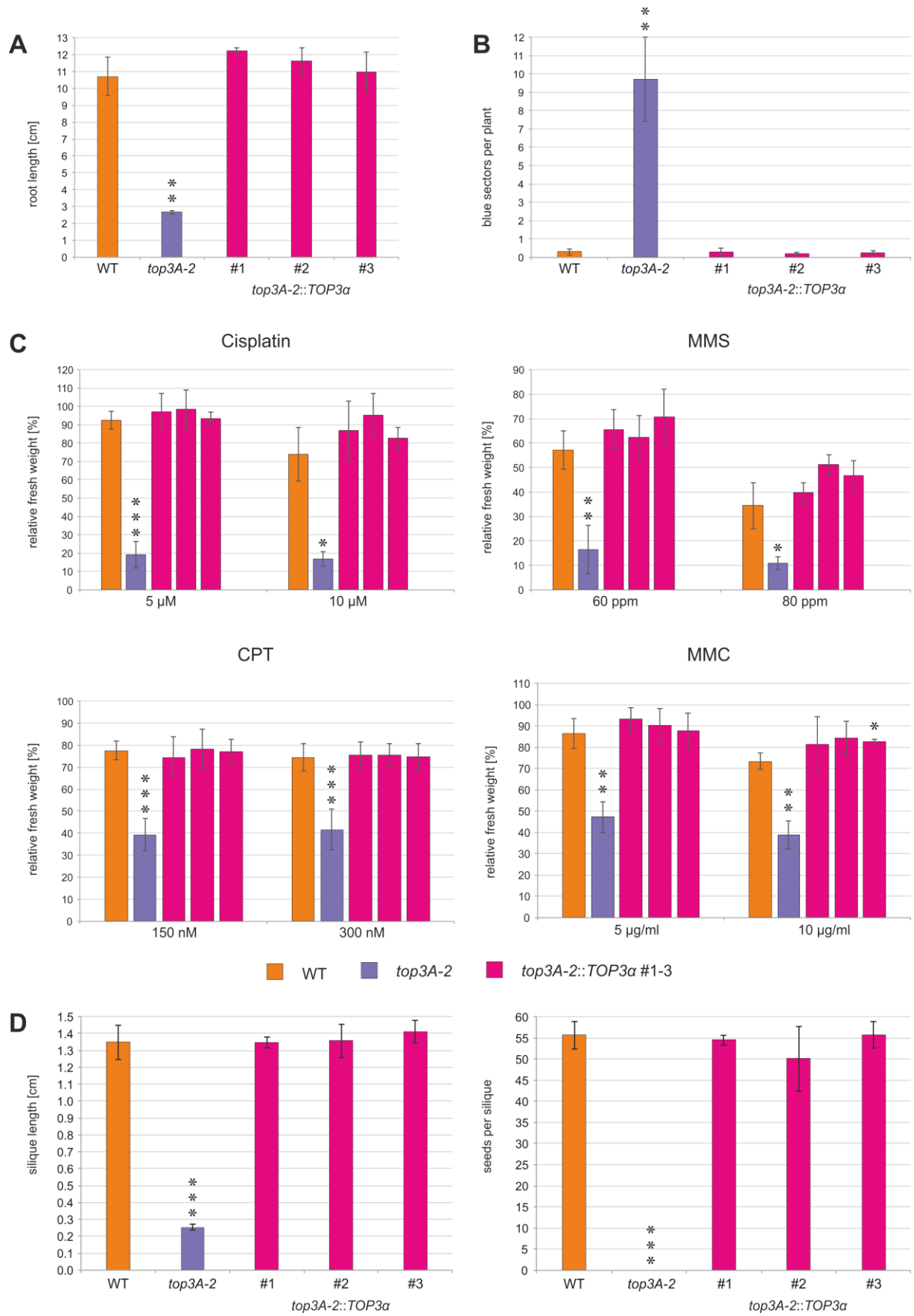

Supplement: S6 Fig — Root length (A), recombination rate (B), genotoxin sensitivity (C) and fertility (D) of three independent top3A-2::TOP3α lines was determined in comparison to the top3A-2 mutant and wild type (WT) plants. The complementation lines exhibited a complete reversal of all mutant phenotypes. Significant differences to the WT control were calculated using a two-tailed t-test with unequal variances: * p < 0.05, ** p < 0.01, *** p < 0.001. (PDF) [file pgen.1007674.s006.pdf]

**A**

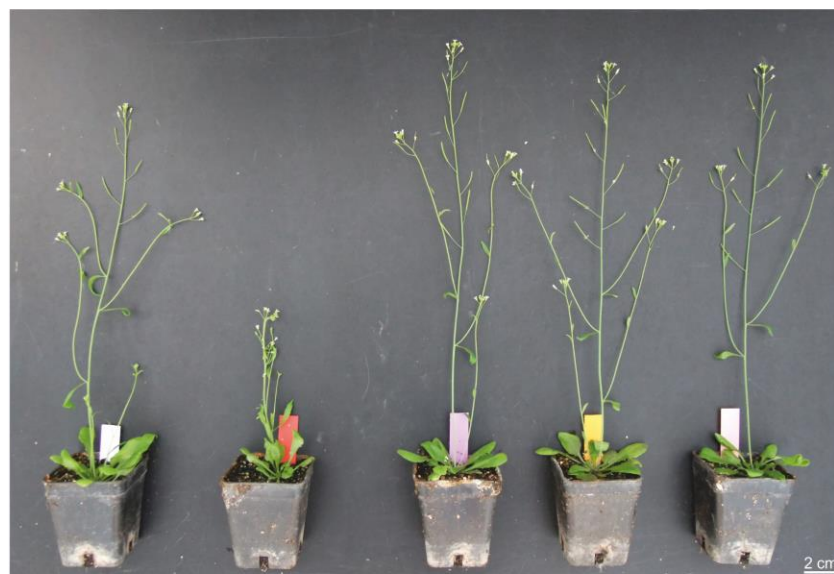

WT

*top3A-6*

#1

#2

#3

*top3A-6::TOP3α*

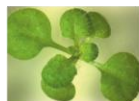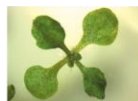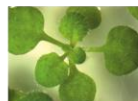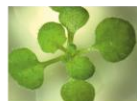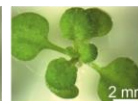

**B**

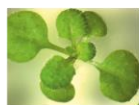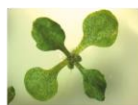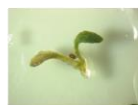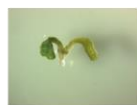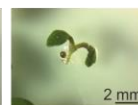

WT

*top3A-6*

#1

#2

#3

*top3A-6::TOP3α-Central*

**C**

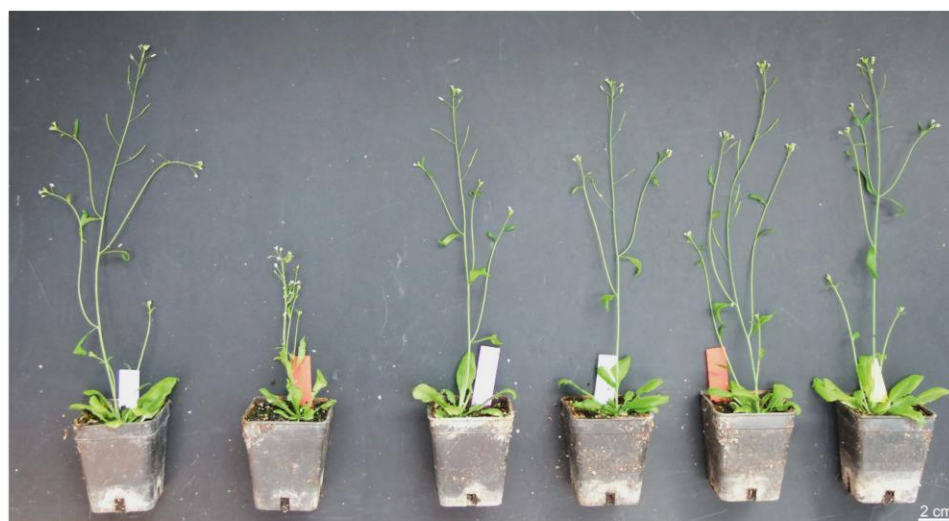

WT

*top3A-6*

#1

#2

#3

#4

*top3A-6::TOP3α-N-Term*

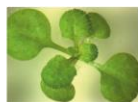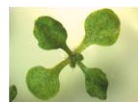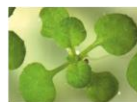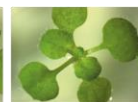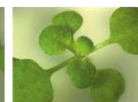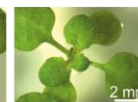

Supplement: S7 Fig — (A) Two-week-old seedlings and five-week-old plants from three individual top3A-6::TOP3α complementation lines compared to top3A-6 mutants and wild type (WT) plants. The characteristic growth defects of top3A-6 could be fully complemented by expression of TOP3α in all three complementation lines, leading to a growth phenotype indistinguishable to WT plants. (B) Two-week-old plantlets of three individual top3A-6::TOP3α-Central complementation lines compared to top3A-6 mutants and WT plants. While top3A-6 mutant lines exhibit characteristic growth defects with dark and deformed leaves, expression of TOP3α-Central in this line leads to an enhanced growth defect. Plants feature only the cotyledons that are deformed and no roots are formed. (C) Two-week-old plantlets and five-week-old plants of four individual top3A-6::TOP3α-N-Term complementation lines are compared to top3A-6 mutants and WT plants. The growth defects of top3A-6 mutant plants could be complemented completely by expression of TOP3α-N-Term leading to plants indistinguishable from the WT. (PDF) [file pgen.1007674.s007.pdf]

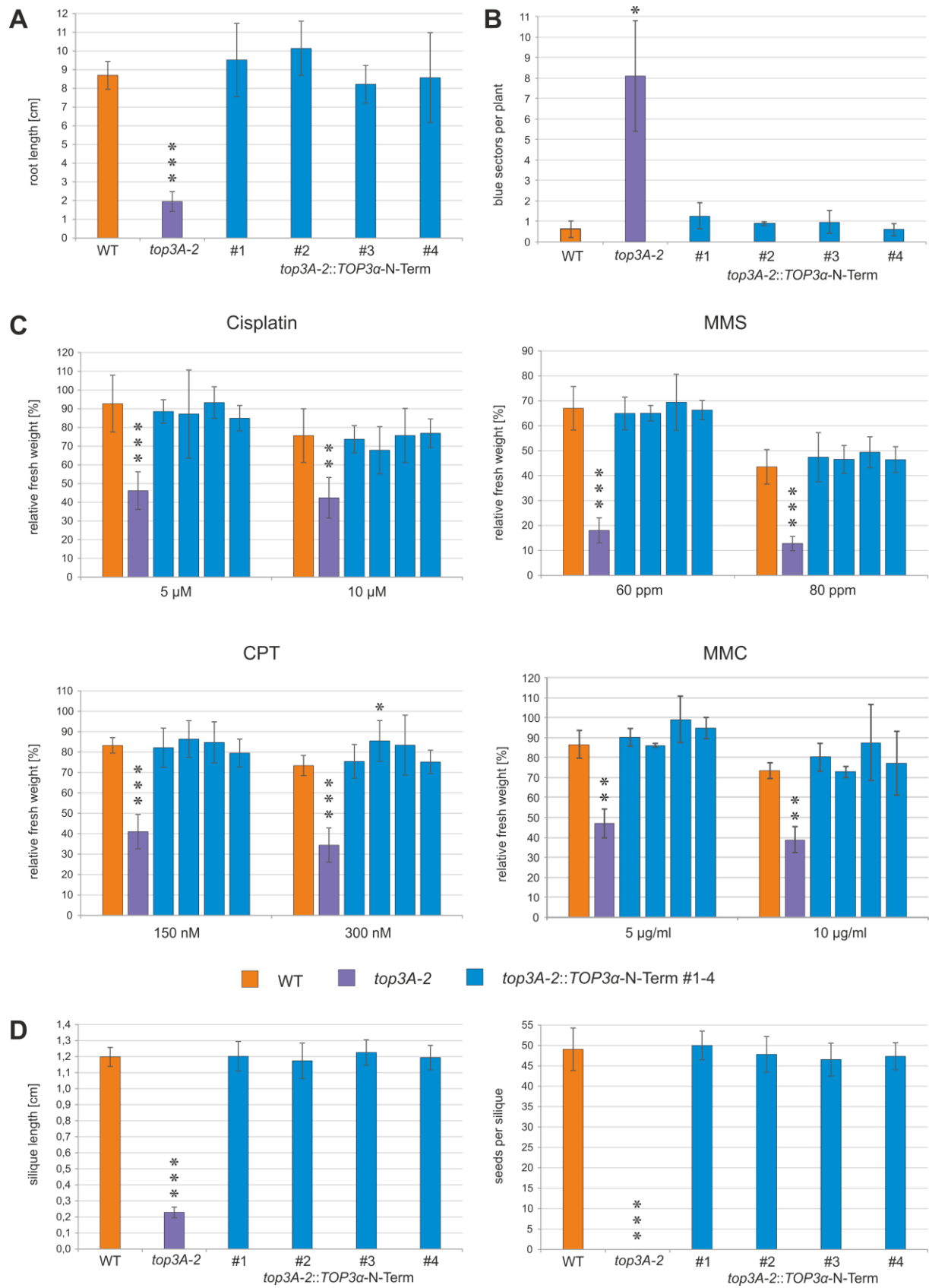

Supplement: S8 Fig — Root length (A), recombination rate (B), genotoxin sensitivity (C) and fertility (D) of four independent top3A-2::TOP3α-N-Term lines was determined in comparison to the top3A-2 mutant and wild type (WT) plants. The complementation lines exhibited a complete reversal of all mutant phenotypes. Significant differences to the WT control were calculated using a two-tailed t-test with unequal variances: * p < 0.05, ** p < 0.01, *** p < 0.001. (PDF) [file pgen.1007674.s008.pdf]

**A**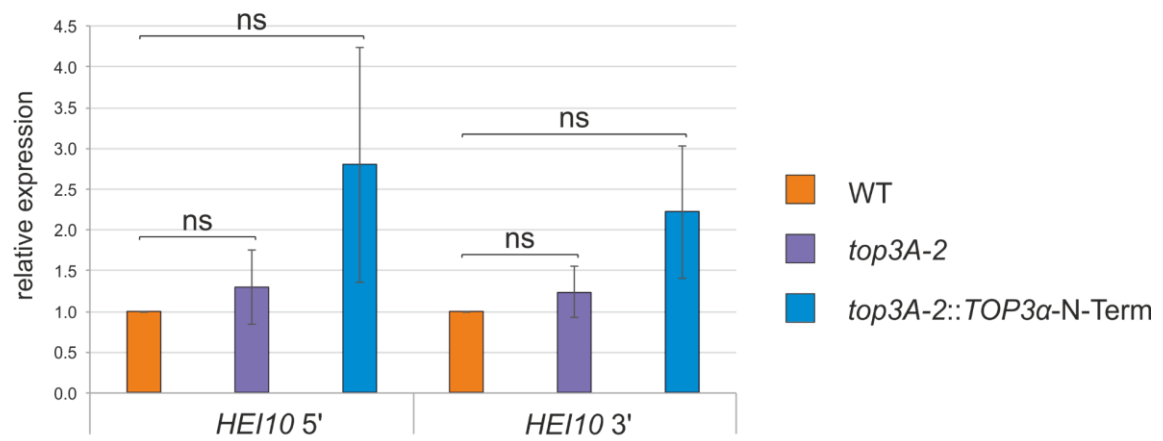**B**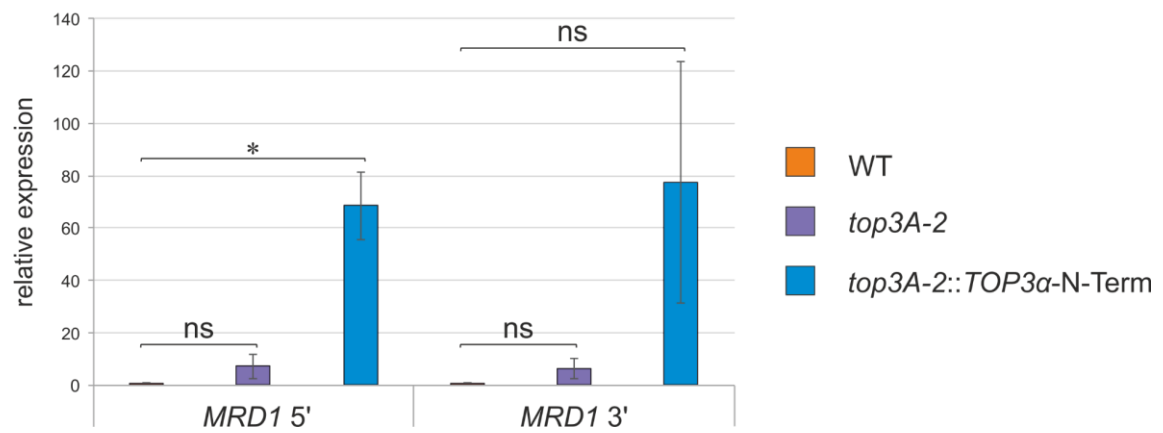

Supplement: S9 Fig — The expression of HEI10 and MRD1 in top3A-2, top3A-2::TOP3α-N-Term and wild type (WT) plants was tested by qRT-PCR analysis with two primer pairs each. Three independent assays were performed and mean values with standard deviation (error bars) were calculated. Statistical differences were calculated using a two-tailed t-test with unequal variances: * p < 0.05, ns = not significant. (A) For HEI10, the expression at the 5’ and 3’ end of the gene was comparable in all analysed lines. (B) For MRD1, the expression at the 5’ end of the gene was significantly increased in top3A-2::TOP3α-N-Term (69x) compared to the WT. (PDF) [file pgen.1007674.s009.pdf]

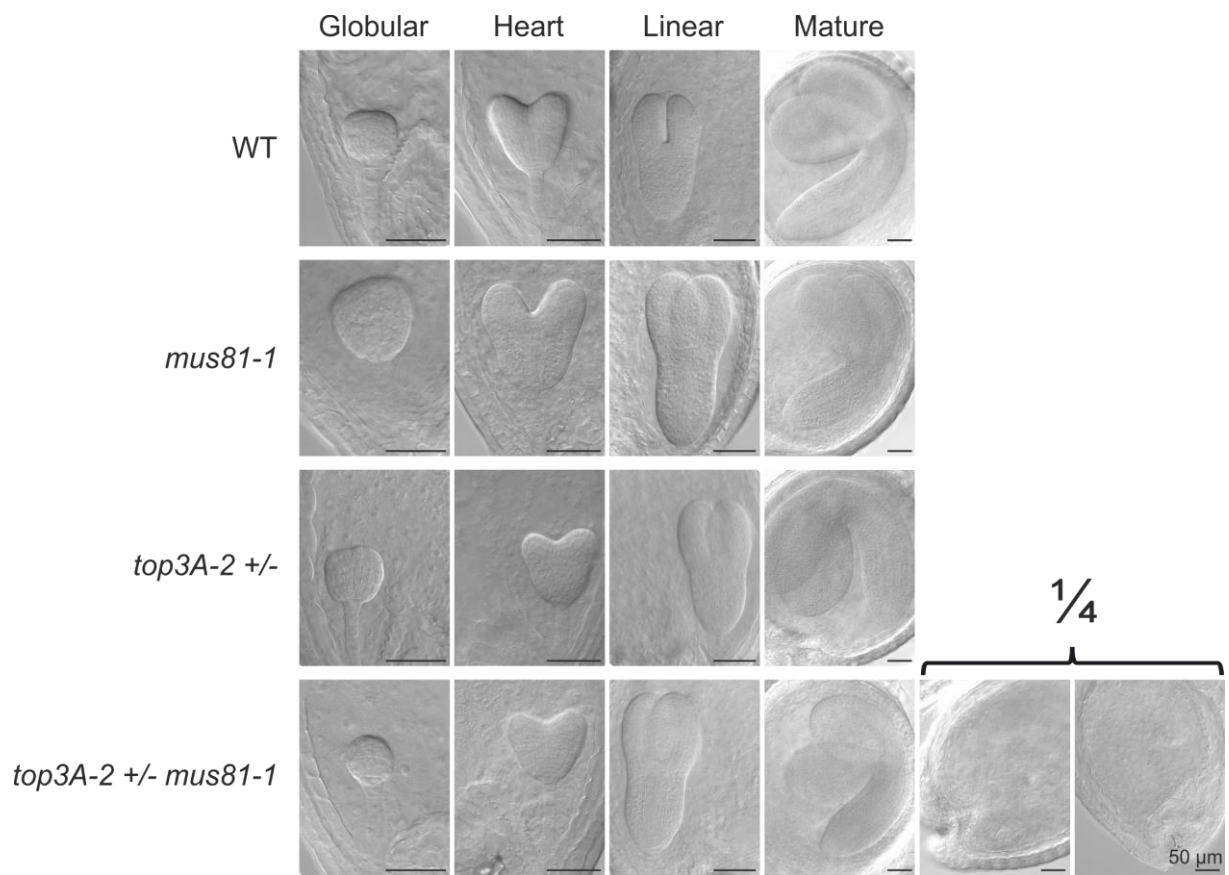

Supplement: S10 Fig — Depicted are representative embryos of exemplary top3A-2 +/- mus81-1 double mutants compared to wild type (WT), top3A-2 +/- and mus81-1 embryos. All lines showed complete embryo development leading to mature embryos. In heterozygous top3A-2 mus81-1 double mutant lines, a Χ2 test confirmed a ratio of ¼ seeds with lacking or deformed embryos, corresponding to the amount of homozygous top3A-2 mus81-1 double mutants. (PDF) [file pgen.1007674.s010.pdf]
