# Supplementary material for: The topoisomerase 3α zinc-finger domain T1 of Arabidopsis thaliana is required for targeting the enzyme activity to Holliday junction-like DNA repair intermediates
Source: PLoS Genet. 2018 Sep 17;14(9):e1007674. doi: 10.1371/journal.pgen.1007674 (PMC6160208; doi:10.1371/journal.pgen.1007674)
Supplement: S2 Fig — cDNA sequences of CRISPR/Cas9 induced top3α mutant lines were aligned with wild type (WT) sequences. The start codon is depicted in blue, sequences differing from the WT in red. All mutations from the different mutant lines lead to a frameshift, generating a premature stop codon (red box). (PDF) [file pgen.1007674.s002.pdf]

```
                                                                                                                      STOP
WT          1 ATGTCGCGACG-AGGCGGTGGCCCCGTGACAGTGTTGAACGTGGCGGAGAAGCCGTCCGTGGCGAAGTCAGTGGCGGGGATTCTATCCCGTGGAACTTTCCGGACTCGGGAGGGTAGGTC 119
top3A-3       ATGTCGCGACGTAGGCGGTGGCCCCGTGACAGTGTTGAACGTGGCGGAGAAGCCGTCCGTGGCGAAGTCAGTGGCGGGGATTCTATCCCGTGGAACTTTCCGGACTCGGGAGGGTAGGTC
top3A-4       ATGTCGCG------------------TGACAGTGTTGAACGTGGCGGAGAAGCCGTCCGTGGCGAAGTCAGTGGCGGGGATTCTATCCCGTGGAACTTTCCGGACTCGGGAGGGTAGGTC
top3A-5       ATGTCGCGACG-AGGCGGTGGCCCCGTGACAGTGTTGAACGTGGCGGAGAAGCCGTCCGTGGCGAAGTCAGTGGCGGGGATTCTATCCCGTGGAACTTTCCGGACTCGGGAGGGTAGGTC
top3A-6       ATGTCGCGACG-AGGCGGTGGCCCCGTGACAGTGTTGAACGTGGCGGAGAAGCCGTCCGTGGCGAAGTCAGTGGCGGGGATTCTATCCCGTGGAACTTTCCGGACTCGGGAGGGTAGGTC

WT        120 TCGGTACAACAAAATCTTCGAGTTCGATTACGCCATCAATGGGCAGCCGTGTCGTATGCTAATGACCTCCGTCATCGGCCACCTGATGGAGCTTGAGTTCGCCGATCGTTACCGCAAATG 239
top3A-3       TCGGTACAACAAAATCTTCGAGTTCGATTACGCCATCAATGGGCAGCCGTGTCGTATGCTAATGACCTCCGTCATCGGCCACCTGATGGAGCTTGAGTTCGCCGATCGTTACCGCAAATG
top3A-4       TCGGTACAACAAAATCTTCGAGTTCGATTACGCCATCAATGGGCAGCCGTGTCGTATGCTAATGACCTCCGTCATCGGCCACCTGATGGAGCTTGAGTTCGCCGATCGTTACCGCAAATG
top3A-5       TCGGTACAACAAAATCTTCGAGTTCGATTACGCCATCAATGGGCAGCCGTGTCGTATGCTAATGACCTCCGTCATCGGCCACCTGATGGAGCTTGAGTTCGCCGATCGTTACCGCAAATG
top3A-6       TCGGTACAACAAAATCTTCGAGTTCGATTACGCCATCAATGGGCAGCCGTGTCGTATGCTAATGACCTCCGTCATCGGCCACCTGATGGAGCTTGAGTTCGCCGATCGTTACCGCAAATG

                                        STOP                             Exon 1|Exon 2       STOP
WT        240 GCACTCCTGCGACCCGGCAGATCTGTACC-AAGCTCCGGTCATGAAACACGTTCCCGAG|GACAAAAAGGATATTAA 314
top3A-3       GCACTCCTGCGACCCGGCAGATCTGTACC-AAGCTCCGGTCATGAAACACGTTCCCGAG|GACAAAAAGGATATTAA
top3A-4       GCACTCCTGCGACCCGGCAGATCTGTACC-AAGCTCCGGTCATGAAACACGTTCCCGAG|GACAAAAAGGATATTAA
top3A-5       GCACTCCTGCGACCCGGCAGATCTGTACCAAAGCTCCGGTCATGAAACACGTTCCCGAG|GACAAAAAGGATATTAA
top3A-6       GCACTCCTGC---------------TAAT-GACCTCCGGTCATGAAACACGTTCCCGAG|GACAAAAAGGATATTAA
```
