# Supplementary material for: The topoisomerase 3α zinc-finger domain T1 of Arabidopsis thaliana is required for targeting the enzyme activity to Holliday junction-like DNA repair intermediates
Source: PLoS Genet. 2018 Sep 17;14(9):e1007674. doi: 10.1371/journal.pgen.1007674 (PMC6160208; doi:10.1371/journal.pgen.1007674)
Supplement: S5 Fig — Six-week-old wild type (WT) plants containing the different TOP3α complementation constructs TOP3α-ΔTOPRIM, TOP3α-Y342F, TOP3α-Central (A) and TOP3α-N-Term, TOP3α-ΔZnFT1, TOP3α-ΔZnFCCHC1, TOP3α-ΔZnFGRF, TOP3α-ΔZnFCCHC2 (B) are shown in comparison to the WT. All complementation lines exhibit a growth phenotype indistinguishable from the WT. (PDF) [file pgen.1007674.s005.pdf]

A

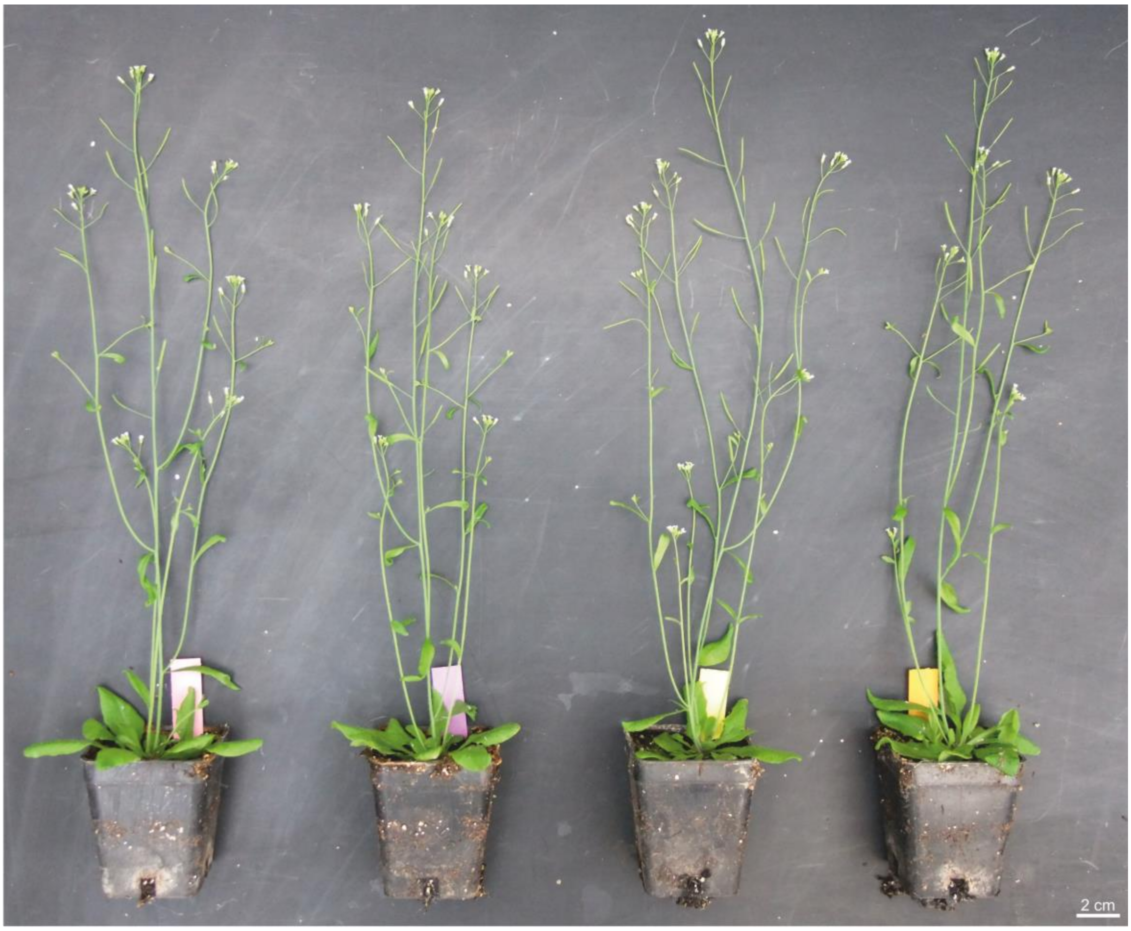

WT | WT::*TOP3α*-ΔTOPRIM | WT::*TOP3α*-Y342F | WT::*TOP3α*-Central

B

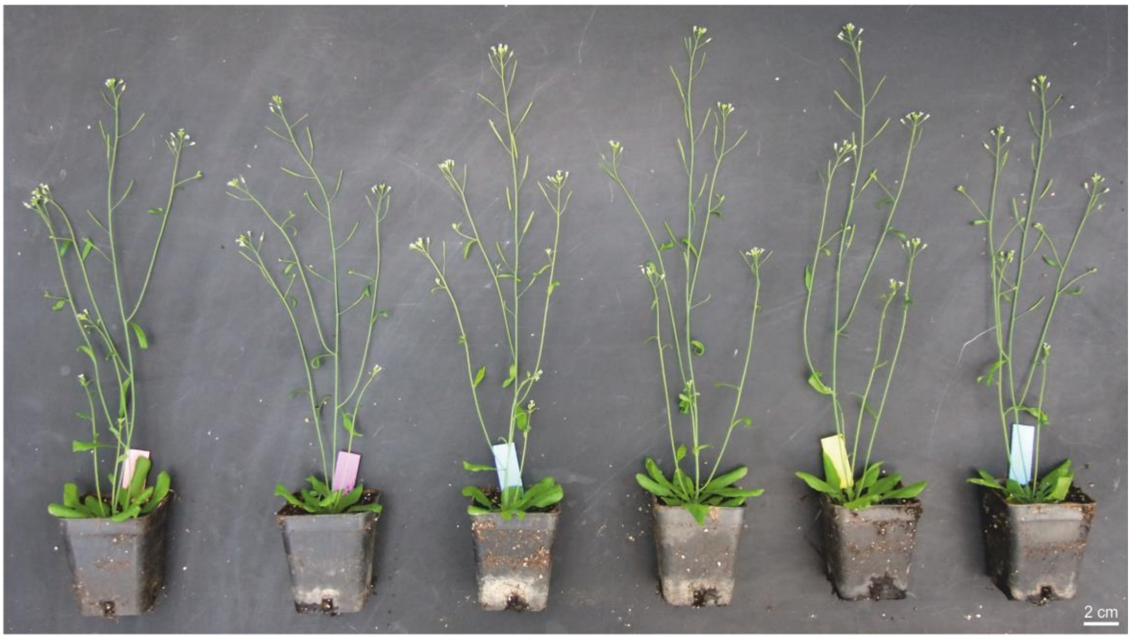

WT | WT::*TOP3α*-N-Term | WT::*TOP3α*-ΔZnFT1 | WT::*TOP3α*-ΔZnFCCHC1 | WT::*TOP3α*-ΔZnFGRF | WT::*TOP3α*-ΔZnFCCHC2
