# Supplementary material for: The topoisomerase 3α zinc-finger domain T1 of Arabidopsis thaliana is required for targeting the enzyme activity to Holliday junction-like DNA repair intermediates
Source: PLoS Genet. 2018 Sep 17;14(9):e1007674. doi: 10.1371/journal.pgen.1007674 (PMC6160208; doi:10.1371/journal.pgen.1007674)
Supplement: S11 Fig — Depicted is an alignment of the gDNA sequence from TOP3α exon 15 of top3A-1 and the wild type (WT) sequence. Sequences differing from the WT are depicted in red. In top3A-1, a 44 bp deletion was identified on mRNA level leading to a premature stop codon in frame (red box). On protein level, this results in a truncated TOP3α protein, missing all C-terminal zinc-finger domains and the last 21 amino acids of the central domain. (PDF) [file pgen.1007674.s011.pdf]

```
WT        1686 GTATGAACTTTGGAAACCAAATCTCAGAGCCCTTATGGAACATGATATGA 1735
top3A-1        GTATGAACTTTGGAAACCAAATCTCAGAGCC-------------------

WT        1736 ATGAAGTTAGCGTTGGCAGGAAGACCAAAGCTGAAGTTCTTGAAACATGT 1785
top3A-1        -------------------------CAAAGCTGAAGTTCTTGAAACATGT
                                               STOP

WT        1786 TTGCAGCAAATGAAAGCTTGTTTCTTAGAT 1815
top3A-1        TTGCAGCAAATGAAAGCTTGTTTCTTAGAT
```
