# Supplementary material for: The topoisomerase 3α zinc-finger domain T1 of Arabidopsis thaliana is required for targeting the enzyme activity to Holliday junction-like DNA repair intermediates
Source: PLoS Genet. 2018 Sep 17;14(9):e1007674. doi: 10.1371/journal.pgen.1007674 (PMC6160208; doi:10.1371/journal.pgen.1007674)
Supplement: S1 Table — (PDF) [file pgen.1007674.s012.pdf]

S1 Table: Statistical analysis of embryo development in *top3A-6* +/- ::*TOP3α*-Y342F/ΔTOPRIM.

Raw data from embryo analyses in three independent *top3A-6* +/- ::*TOP3α*-Y342F/ΔTOPRIM lines, *top3A-6* +/- and the wild type (WT) is depicted. Whether the number of seeds containing a deformed or lacking embryo corresponded to a ratio of ¼ or not was determined using a X^2-test.

Genotype		intact embryo	lacking/deformed embryo	total number of seeds	expectation intact embryo	expectation lacking/deformed embryo	$B_i^2/E_{i\ pos}$	$B_i^2/E_{i\ neg}$	χ^2	1/4 of seeds containing deformed/lacking embryo
**WT**	Silique 1	29	2	31	23.25	7.8	36.2	0.5	5.7	no
	Silique 2	40	2	42	31.5	10.5	50.8	0.4	9.2	no
	Silique 3	42	1	43	32.25	10.8	54.7	0.1	11.8	no
	Silique 4	34	0	34	25.5	8.5	45.3	0.0	11.3	no
	Silique 5	26	0	26	19.5	6.5	34.7	0.0	8.7	no
***top3A-6* +/-**	Silique 1	36	2	38	28.5	9.5	45.5	0.4	7.9	no
	Silique 2	30	1	31	23.25	7.8	38.7	0.1	7.8	no
	Silique 3	43	2	45	33.75	11.3	54.8	0.4	10.1	no
	Silique 4	38	4	42	31.5	10.5	45.8	1.5	5.4	no
	Silique 5	29	0	29	21.75	7.3	38.7	0.0	9.7	no
***top3A-6* +/- ::*TOP3α*-Y342F #1**	Silique 1	31	11	42	31.5	10.5	30.5	11.5	0.0	yes
	Silique 2	30	13	43	32.25	10.8	27.9	15.7	0.6	yes
	Silique 3	19	11	30	22.5	7.5	16.0	16.1	2.2	yes
	Silique 4	31	11	42	31.5	10.5	30.5	11.5	0.0	yes
	Silique 5	30	12	42	31.5	10.5	28.6	13.7	0.3	yes
***top3A-6* +/- ::*TOP3α*-Y342F #2**	Silique 1	21	9	30	22.5	7.5	19.6	10.8	0.4	yes
	Silique 2	29	10	39	29.25	9.8	28.8	10.3	0.0	yes
	Silique 3	16	7	23	17.25	5.8	14.8	8.5	0.4	yes
	Silique 4	34	12	46	34.5	11.5	33.5	12.5	0.0	yes
	Silique 5	25	9	34	25.5	8.5	24.5	9.5	0.0	yes
***top3A-6* +/- ::*TOP3α*-Y342F #3**	Silique 1	15	9	24	18	6.0	12.5	13.5	2.0	yes
	Silique 2	15	8	23	17.25	5.8	13.0	11.1	1.2	yes
	Silique 3	12	6	18	13.5	4.5	10.7	8.0	0.7	yes
	Silique 4	14	8	22	16.5	5.5	11.9	11.6	1.5	yes
	Silique 5	18	7	25	18.75	6.3	17.3	7.8	0.1	yes
***top3A-6* +/- ::*TOP3α*-ΔTOPRIM #1**	Silique 1	30	17	47	35.25	11.8	25.5	24.6	3.1	yes
	Silique 2	29	8	37	27.75	9.3	30.3	6.9	0.2	yes
	Silique 3	23	12	35	26.25	8.8	20.2	16.5	1.6	yes
	Silique 4	26	12	38	28.5	9.5	23.7	15.2	0.9	yes
	Silique 5	33	8	41	30.75	10.3	35.4	6.2	0.7	yes
***top3A-6* +/- ::*TOP3α*-ΔTOPRIM #2**	Silique 1	28	10	38	28.5	9.5	27.5	10.5	0.0	yes
	Silique 2	30	13	43	32.25	10.8	27.9	15.7	0.6	yes
	Silique 3	31	14	45	33.75	11.3	28.5	17.4	0.9	yes
	Silique 4	27	10	37	27.75	9.3	26.3	10.8	0.1	yes
	Silique 5	28	15	43	32.25	10.8	24.3	20.9	2.2	yes
***top3A-6* +/- ::*TOP3α*-ΔTOPRIM #3**	Silique 1	13	4	17	12.75	4.3	13.3	3.8	0.0	yes
	Silique 2	30	12	42	31.5	10.5	28.6	13.7	0.3	yes
	Silique 3	25	11	36	27	9.0	23.1	13.4	0.6	yes
	Silique 4	19	7	26	19.5	6.5	18.5	7.5	0.1	yes
	Silique 5	31	12	43	32.25	10.8	29.8	13.4	0.2	yes

$\chi^2 < \chi^2_{tab}$ (1;0,95) = 3,84
