# Supplementary material for: The topoisomerase 3α zinc-finger domain T1 of Arabidopsis thaliana is required for targeting the enzyme activity to Holliday junction-like DNA repair intermediates
Source: PLoS Genet. 2018 Sep 17;14(9):e1007674. doi: 10.1371/journal.pgen.1007674 (PMC6160208; doi:10.1371/journal.pgen.1007674)
Supplement: S2 Table — (PDF) [file pgen.1007674.s013.pdf]

S2 Table: Statistical analysis of embryo development in *top3A-2* +/- ::*TOP3α*-Y342F/ΔTOPRIM.

Raw data from embryo analyses in three independent *top3A-2* +/- ::*TOP3α*-Y342F/ΔTOPRIM lines, *top3A-2* +/- and the wild type (WT) is depicted. Whether the number of seeds containing a deformed or lacking embryo corresponded to a ratio of ¼ or not was determined using a X^2-test.

Genotype		intact embryo	lacking/deformed embryo	total number of seeds	expectation intact embryo	expectation lacking/deformed embryo	$B_i^2/E_{i\,pos}$	$B_i^2/E_{i\,neg}$	χ^2	1/4 of seeds containing deformed/lacking embryo
**WT**	Silique 1	30	0	30	22.5	7.5	40.0	0.0	10.0	no
	Silique 2	40	0	40	30	10.0	53.3	0.0	13.3	no
	Silique 3	43	2	45	33.75	11.3	54.8	0.4	10.1	no
	Silique 4	35	3	38	28.5	9.5	43.0	0.9	5.9	no
	Silique 5	34	0	34	25.5	8.5	45.3	0.0	11.3	no
top3A-2 +/-	Silique 1	37	1	38	28.5	9.5	48.0	0.1	10.1	no
	Silique 2	33	0	33	24.75	8.3	44.0	0.0	11.0	no
	Silique 3	28	3	31	23.25	7.8	33.7	1.2	3.9	no
	Silique 4	45	3	48	36	12.0	56.3	0.8	9.0	no
	Silique 5	31	3	34	25.5	8.5	37.7	1.1	4.7	no
***top3A-2 +/-* ::*TOP3α*-Y342F #1**	Silique 1	31	12	43	32.25	10.8	29.8	13.4	0.2	yes
	Silique 2	43	16	59	44.25	14.8	41.8	17.4	0.1	yes
	Silique 3	35	14	49	36.75	12.3	33.3	16.0	0.3	yes
	Silique 4	44	14	58	43.5	14.5	44.5	13.5	0.0	yes
	Silique 5	31	14	45	33.75	11.3	28.5	17.4	0.9	yes
***top3A-2 +/-* ::*TOP3α*-Y342F #2**	Silique 1	22	14	36	27	9.0	17.9	21.8	3.7	yes
	Silique 2	38	12	50	37.5	12.5	38.5	11.5	0.0	yes
	Silique 3	27	7	34	25.5	8.5	28.6	5.8	0.4	yes
	Silique 4	21	13	34	25.5	8.5	17.3	19.9	3.2	yes
	Silique 5	28	12	40	30	10.0	26.1	14.4	0.5	yes
***top3A-2 +/-* ::*TOP3α*-Y342F #3**	Silique 1	36	15	51	38.25	12.8	33.9	17.6	0.5	yes
	Silique 2	32	12	44	33	11.0	31.0	13.1	0.1	yes
	Silique 3	34	17	51	38.25	12.8	30.2	22.7	1.9	yes
	Silique 4	23	7	30	22.5	7.5	23.5	6.5	0.0	yes
	Silique 5	24	10	34	25.5	8.5	22.6	11.8	0.4	yes
***top3A-2 +/-* ::*TOP3α*-ΔTOPRIM #1**	Silique 1	24	10	34	25.5	8.5	22.6	11.8	0.4	yes
	Silique 2	36	12	48	36	12.0	36.0	12.0	0.0	yes
	Silique 3	32	9	41	30.75	10.3	33.3	7.9	0.2	yes
	Silique 4	29	14	43	32.25	10.8	26.1	18.2	1.3	yes
	Silique 5	33	8	41	30.75	10.3	35.4	6.2	0.7	yes
***top3A-2 +/-* ::*TOP3α*-ΔTOPRIM #2**	Silique 1	25	7	32	24	8.0	26.0	6.1	0.2	yes
	Silique 2	28	9	37	27.75	9.3	28.3	8.8	0.0	yes
	Silique 3	24	14	38	28.5	9.5	20.2	20.6	2.8	yes
	Silique 4	13	7	20	15	5.0	11.3	9.8	1.1	yes
	Silique 5	23	7	30	22.5	7.5	23.5	6.5	0.0	yes
***top3A-2 +/-* ::*TOP3α*-ΔTOPRIM #3**	Silique 1	35	18	53	39.75	13.3	30.8	24.5	2.3	yes
	Silique 2	13	5	18	13.5	4.5	12.5	5.6	0.1	yes
	Silique 3	42	23	65	48.75	16.3	36.2	32.6	3.7	yes
	Silique 4	42	16	58	43.5	14.5	40.6	17.7	0.2	yes
	Silique 5	40	13	53	39.75	13.3	40.3	12.8	0.0	yes

$\chi^2 < \chi^2_{tab}$ (1;0.95) = 3.84
