# Supplementary material for: The topoisomerase 3α zinc-finger domain T1 of Arabidopsis thaliana is required for targeting the enzyme activity to Holliday junction-like DNA repair intermediates
Source: PLoS Genet. 2018 Sep 17;14(9):e1007674. doi: 10.1371/journal.pgen.1007674 (PMC6160208; doi:10.1371/journal.pgen.1007674)
Supplement: S3 Table — (PDF) [file pgen.1007674.s014.pdf]

S3 Table: Differentially expressed genes in *top3A-2* and *top3A-2*::*TOP3α*-N-Term.
Differentially expressed genes from *top3A-2* mutants and *top3A-2*::*TOP3α*-N-Term complementation lines compared to wild type (WT) plants.

**WT vs. *top3A-2***	**WT vs. *top3A-2*::*TOP3α*-N-Term**
AT1G03710	AT1G53480
AT1G04360	AT1G53490
AT1G04610	AT3G30720
AT1G05250	AT5G63920
AT1G05260	
AT1G05310	
AT1G05490	
AT1G05650	
AT1G05660	
AT1G05710	
AT1G06120	
AT1G06350	
AT1G06830	
AT1G07135	
AT1G07440	
AT1G07500	
AT1G08090	
AT1G08260	
AT1G09240	
AT1G09250	
AT1G10050	
AT1G10470	
AT1G12040	
AT1G12080	
AT1G12200	
AT1G12740	
AT1G12890	
AT1G12950	
AT1G13330	
AT1G13650	
AT1G13710	
AT1G14120	
AT1G14160	
AT1G14250	
AT1G14630	
AT1G14790	
AT1G15310	
AT1G15460	
AT1G17300	
AT1G17460	
AT1G17590	
AT1G17960	
AT1G18100	

AT1G18970
AT1G19050
AT1G19900
AT1G20180
AT1G20450
AT1G20750
AT1G21100
AT1G21440
AT1G21528
AT1G22210
AT1G22250
AT1G23340
AT1G23540
AT1G24095
AT1G26240
AT1G26250
AT1G26450
AT1G26590
AT1G26945
AT1G27740
AT1G27890
AT1G29025
AT1G29195
AT1G29420
AT1G29980
AT1G30040
AT1G30160
AT1G30170
AT1G30660
AT1G30750
AT1G30760
AT1G30870
AT1G30950
AT1G31290
AT1G31710
AT1G31950
AT1G32510
AT1G32860
AT1G33340
AT1G33560
AT1G33790
AT1G35720
AT1G35910
AT1G36180
AT1G43160
AT1G43910
AT1G44010

AT1G44830
AT1G46480
AT1G47960
AT1G49360
AT1G49860
AT1G49920
AT1G51900
AT1G52120
AT1G52700
AT1G53480
AT1G53490
AT1G54020
AT1G54160
AT1G54280
AT1G55210
AT1G55990
AT1G56600
AT1G58370
AT1G59660
AT1G59740
AT1G60030
AT1G60500
AT1G60750
AT1G60989
AT1G61470
AT1G61590
AT1G61710
AT1G62262
AT1G62290
AT1G62360
AT1G62540
AT1G62560
AT1G62730
AT1G62980
AT1G63210
AT1G63440
AT1G63550
AT1G63750
AT1G64370
AT1G64540
AT1G64590
AT1G65150
AT1G65310
AT1G65370
AT1G66040
AT1G66100
AT1G66480

AT1G66520
AT1G67760
AT1G67810
AT1G68880
AT1G69260
AT1G69310
AT1G69526
AT1G70260
AT1G70440
AT1G70850
AT1G71770
AT1G71960
AT1G72830
AT1G73040
AT1G73220
AT1G73290
AT1G73300
AT1G73330
AT1G73680
AT1G74890
AT1G75900
AT1G76180
AT1G76240
AT1G76470
AT1G77330
AT1G77520
AT1G77530
AT1G78090
AT1G78990
AT1G79250
AT1G79760
AT1G80130
AT2G01420
AT2G01520
AT2G01530
AT2G02580
AT2G02610
AT2G03505
AT2G04190
AT2G05160
AT2G05520
AT2G06255
AT2G06420
AT2G14960
AT2G15780
AT2G16005
AT2G16380

AT2G16660
AT2G16970
AT2G16980
AT2G17500
AT2G18190
AT2G18193
AT2G18230
AT2G18600
AT2G18720
AT2G18800
AT2G19970
AT2G20520
AT2G20800
AT2G21220
AT2G21640
AT2G21770
AT2G22122
AT2G22510
AT2G22660
AT2G22800
AT2G23120
AT2G23540
AT2G24255
AT2G24710
AT2G24720
AT2G24762
AT2G24980
AT2G25240
AT2G25370
AT2G25900
AT2G26480
AT2G26820
AT2G27370
AT2G27385
AT2G27535
AT2G27550
AT2G28210
AT2G28500
AT2G28671
AT2G29130
AT2G31083
AT2G31085
AT2G31141
AT2G31480
AT2G31980
AT2G32280
AT2G32300

AT2G32930
AT2G32960
AT2G33790
AT2G33880
AT2G34170
AT2G34340
AT2G34490
AT2G35380
AT2G36100
AT2G36120
AT2G36261
AT2G36985
AT2G37370
AT2G37750
AT2G38250
AT2G38340
AT2G38380
AT2G38530
AT2G39040
AT2G39350
AT2G39370
AT2G39430
AT2G39650
AT2G39800
AT2G39855
AT2G40230
AT2G40610
AT2G40955
AT2G41300
AT2G41410
AT2G41480
AT2G41730
AT2G42430
AT2G44390
AT2G44800
AT2G45460
AT2G45650
AT2G46530
AT2G46640
AT2G46680
AT2G46850
AT2G46860
AT2G47010
AT2G47180
AT2G47270
AT2G47680
AT2G47780

AT2G47880
AT2G47890
AT2G48090
AT3G01175
AT3G01190
AT3G01345
AT3G01600
AT3G02020
AT3G02810
AT3G02850
AT3G03341
AT3G03480
AT3G03660
AT3G03670
AT3G04330
AT3G05150
AT3G05620
AT3G05650
AT3G05690
AT3G05890
AT3G06390
AT3G07540
AT3G07800
AT3G08040
AT3G08590
AT3G08860
AT3G08990
AT3G09070
AT3G09260
AT3G09390
AT3G10190
AT3G10290
AT3G10600
AT3G11385
AT3G11550
AT3G12240
AT3G12410
AT3G12700
AT3G12830
AT3G13090
AT3G13130
AT3G13404
AT3G13784
AT3G13790
AT3G14060
AT3G14225
AT3G14450

AT3G14530
AT3G14540
AT3G14550
AT3G15170
AT3G15650
AT3G15720
AT3G16330
AT3G16360
AT3G16410
AT3G16450
AT3G17690
AT3G18280
AT3G18610
AT3G19550
AT3G20100
AT3G20380
AT3G20470
AT3G20940
AT3G21310
AT3G21460
AT3G21720
AT3G21770
AT3G21850
AT3G22060
AT3G22142
AT3G22550
AT3G22600
AT3G22620
AT3G22850
AT3G23470
AT3G23510
AT3G24020
AT3G24450
AT3G24715
AT3G25820
AT3G25830
AT3G25930
AT3G26590
AT3G26820
AT3G27025
AT3G27060
AT3G27070
AT3G27250
AT3G27400
AT3G27620
AT3G27630
AT3G27870

AT3G28500
AT3G28580
AT3G28600
AT3G29250
AT3G30350
AT3G30720
AT3G32030
AT3G42640
AT3G43270
AT3G44326
AT3G44800
AT3G45700
AT3G45710
AT3G46370
AT3G47750
AT3G48100
AT3G48770
AT3G49330
AT3G49960
AT3G50030
AT3G50440
AT3G50640
AT3G50760
AT3G50930
AT3G51560
AT3G51570
AT3G51910
AT3G52115
AT3G52310
AT3G52780
AT3G52790
AT3G53040
AT3G53230
AT3G54040
AT3G54390
AT3G54510
AT3G54580
AT3G54590
AT3G54720
AT3G54770
AT3G54820
AT3G55710
AT3G55720
AT3G56230
AT3G56350
AT3G56500
AT3G56880

AT3G58270
AT3G59340
AT3G59370
AT3G59670
AT3G60280
AT3G60470
AT3G60890
AT3G61090
AT3G61990
AT3G62270
AT3G62680
AT3G62740
AT3G62760
AT3G62950
AT3G62990
AT3G63160
AT3G63240
AT4G00680
AT4G01060
AT4G01140
AT4G01360
AT4G01390
AT4G01420
AT4G01760
AT4G01985
AT4G02110
AT4G02270
AT4G02280
AT4G02330
AT4G02390
AT4G02700
AT4G02830
AT4G02850
AT4G04840
AT4G05170
AT4G05370
AT4G05380
AT4G08400
AT4G08410
AT4G08570
AT4G08867
AT4G10640
AT4G11210
AT4G11320
AT4G11350
AT4G11480
AT4G11650

AT4G11880
AT4G12500
AT4G12510
AT4G12520
AT4G12980
AT4G13300
AT4G13390
AT4G13420
AT4G13480
AT4G13580
AT4G13620
AT4G13820
AT4G14020
AT4G14630
AT4G14650
AT4G14695
AT4G15270
AT4G15290
AT4G15320
AT4G15340
AT4G15390
AT4G15393
AT4G16240
AT4G16920
AT4G17220
AT4G17710
AT4G17980
AT4G18970
AT4G19810
AT4G19840
AT4G20390
AT4G21070
AT4G21650
AT4G21830
AT4G21910
AT4G22230
AT4G22460
AT4G22490
AT4G22666
AT4G22810
AT4G22960
AT4G23680
AT4G24420
AT4G24450
AT4G25220
AT4G25250
AT4G25310

AT4G25330
AT4G25790
AT4G25820
AT4G26010
AT4G26320
AT4G26560
AT4G27150
AT4G27970
AT4G27980
AT4G28170
AT4G28780
AT4G28940
AT4G29140
AT4G29270
AT4G29740
AT4G30250
AT4G30290
AT4G30980
AT4G31330
AT4G31910
AT4G31970
AT4G33120
AT4G33610
AT4G33790
AT4G34000
AT4G34300
AT4G34320
AT4G34580
AT4G34710
AT4G35160
AT4G35190
AT4G36410
AT4G36430
AT4G36700
AT4G37410
AT4G37490
AT4G37900
AT4G37990
AT4G38080
AT4G38210
AT4G38340
AT4G38410
AT4G38460
AT4G38530
AT4G39210
AT4G39500
AT4G39675

AT4G39730
AT4G40090
AT5G01120
AT5G02240
AT5G03780
AT5G04890
AT5G04960
AT5G05500
AT5G05860
AT5G06080
AT5G06200
AT5G06250
AT5G06510
AT5G06630
AT5G06640
AT5G06730
AT5G07310
AT5G07460
AT5G07610
AT5G07760
AT5G08000
AT5G08391
AT5G09300
AT5G09530
AT5G10130
AT5G10230
AT5G10430
AT5G11410
AT5G11460
AT5G12140
AT5G12330
AT5G13170
AT5G13210
AT5G13900
AT5G14020
AT5G14130
AT5G14180
AT5G14490
AT5G15180
AT5G15290
AT5G15380
AT5G15530
AT5G15725
AT5G16240
AT5G16900
AT5G17760
AT5G17980

AT5G18270
AT5G18560
AT5G18690
AT5G18860
AT5G19520
AT5G19550
AT5G20850
AT5G21105
AT5G22460
AT5G22555
AT5G22560
AT5G23010
AT5G23020
AT5G23220
AT5G23360
AT5G24100
AT5G24280
AT5G24420
AT5G25230
AT5G25390
AT5G25610
AT5G26730
AT5G35190
AT5G35940
AT5G37450
AT5G37990
AT5G38020
AT5G38240
AT5G38970
AT5G39110
AT5G40370
AT5G40780
AT5G40790
AT5G40800
AT5G40840
AT5G42050
AT5G42180
AT5G42210
AT5G43330
AT5G45080
AT5G45200
AT5G45350
AT5G45670
AT5G45990
AT5G46260
AT5G46730
AT5G46890

AT5G46900
AT5G47450
AT5G47990
AT5G48020
AT5G48110
AT5G48485
AT5G48720
AT5G49110
AT5G49520
AT5G49850
AT5G51780
AT5G52000
AT5G52050
AT5G52310
AT5G52390
AT5G53120
AT5G53230
AT5G53240
AT5G53250
AT5G53320
AT5G53810
AT5G53830
AT5G54560
AT5G55270
AT5G55490
AT5G56080
AT5G56160
AT5G56490
AT5G56510
AT5G56540
AT5G56630
AT5G57625
AT5G57780
AT5G58390
AT5G58610
AT5G59080
AT5G59090
AT5G59220
AT5G59390
AT5G59510
AT5G60250
AT5G60520
AT5G60660
AT5G61070
AT5G61340
AT5G61490
AT5G61520

AT5G61730
AT5G61740
AT5G61890
AT5G62340
AT5G63660
AT5G63750
AT5G63920
AT5G63990
AT5G64060
AT5G64910
AT5G65990
AT5G66130
AT5G66170
AT5G66390
AT5G66600
AT5G66830
AT5G67400
AT5G67460
