# Supplementary material for: The topoisomerase 3α zinc-finger domain T1 of Arabidopsis thaliana is required for targeting the enzyme activity to Holliday junction-like DNA repair intermediates
Source: PLoS Genet. 2018 Sep 17;14(9):e1007674. doi: 10.1371/journal.pgen.1007674 (PMC6160208; doi:10.1371/journal.pgen.1007674)
Supplement: S4 Table — (PDF) [file pgen.1007674.s015.pdf]

S4 Table: Statistical analysis of embryo development in *top3A-2 +/- mus81-1*.

Raw data from embryo analyses in *top3A-2 +/- mus81-1*, *top3A-2 +/-*, *mus81-1* and the wild type (WT) is depicted. Whether the number of seeds containing a deformed or lacking embryo corresponded to a ratio of ¼ or not was determined using a X^2-test.

Genotype		intact embryo	lacking/deformed embryo	total number of seeds	expectation intact embryo	expectation lacking/deformed embryo	$B_i^2/E_{i\ pos}$	$B_i^2/E_{i\ neg}$	χ^2	1/4 of seeds containing deformed/lacking embryo
**WT**	Silique 1	43	1	44	33	11.0	56.0	0.1	12.1	no
	Silique 2	38	1	39	29.25	9.8	49.4	0.1	10.5	no
	Silique 3	57	0	57	42.75	14.3	76.0	0.0	19.0	no
	Silique 4	37	0	37	27.75	9.3	49.3	0.0	12.3	no
	Silique 5	45	2	47	35.25	11.8	57.4	0.3	10.8	no
mus81-1	Silique 1	52	0	52	39	13.0	69.3	0.0	17.3	no
	Silique 2	45	0	45	33.75	11.3	60.0	0.0	15.0	no
	Silique 3	44	4	48	36	12.0	53.8	1.3	7.1	no
	Silique 4	51	2	53	39.75	13.3	65.4	0.3	12.7	no
	Silique 5	38	4	42	31.5	10.5	45.8	1.5	5.4	no
top3A-2 +/-	Silique 1	48	1	49	36.75	12.3	62.7	0.1	13.8	no
	Silique 2	29	0	29	21.75	7.3	38.7	0.0	9.7	no
	Silique 3	45	0	45	33.75	11.3	60.0	0.0	15.0	no
	Silique 4	50	2	52	39	13.0	64.1	0.3	12.4	no
	Silique 5	37	0	37	27.75	9.3	49.3	0.0	12.3	no
top3A-2 +/- mus81-	Silique 1	33	8	41	30.75	10.3	35.4	6.2	0.7	yes
	Silique 2	27	12	39	29.25	9.8	24.9	14.8	0.7	yes
	Silique 3	46	9	55	41.25	13.8	51.3	5.9	2.2	yes
	Silique 4	30	16	46	34.5	11.5	26.1	22.3	2.3	yes
	Silique 5	24	12	36	27	9.0	21.3	16.0	1.3	yes

$\chi^2 < \chi^2_{tab}$ (1;0,95) = 3,84
