# Supplementary material for: The topoisomerase 3α zinc-finger domain T1 of Arabidopsis thaliana is required for targeting the enzyme activity to Holliday junction-like DNA repair intermediates
Source: PLoS Genet. 2018 Sep 17;14(9):e1007674. doi: 10.1371/journal.pgen.1007674 (PMC6160208; doi:10.1371/journal.pgen.1007674)
Supplement: S5 Table — (PDF) [file pgen.1007674.s016.pdf]

S5 Table: Primer combinations for genotyping.

Line	Primer combination	Product length	Anneal. temp.
top3A-2	T3A-3-1 / T3A-R3A	1200 bp	56 °C
	LB1 / T3A-R3A	800 bp	56 °C
***top3A-2*::*TOP3α*-ΔZnFT1**	T3A-3-1 / ZnFT1-SC-REV	2600 bp	54 °C
	LB1 / T3A-R3A	800 bp	56 °C
***top3A-2*::*TOP3α*-ΔZnFGRF**	T3A-3-1 / ZnFGRF-SC-REV	4000 bp	54 °C
	LB1 / T3A-R3A	800 bp	56 °C
***top3A-2*::*TOP3α*-ΔZnFCCHC1**	T3A-3-1 / ZnFCCHC1-SC-REV	3600 bp	54 °C
	LB1 / T3A-R3A	800 bp	56 °C
***top3A-2*::*TOP3α*-ΔZnFCCHC2**	T3A-3-1 / ZnFCCHC2-SC-REV	4300 bp	58 °C
	LB1 / T3A-R3A	800 bp	56 °C
***top3A-2*::*TOP3α*-ΔTOPRIM**	TOPRIM-SC-FW / T3A-intra-REV	2300 bp	58 °C
	LB1 / T3A-R3A	800 bp	56 °C
***top3A-2*::*TOP3α*-N-Term**	T3A-3-1 / ZnFT1-SC-REV	2600 bp	54 °C
	LB1 / T3A-R3A	800 bp	56 °C
***top3A-2*::*TOP3α*-Central**	T3A-3-1 / ZnFT1-SC-REV	2600 bp	54 °C
	LB1 / T3A-R3A	800 bp	56 °C
top3A-2*::*TOP3α	T3A-3-1 / T3A-nt-REV	4600 bp	54 °C
	LB1 / T3A-R3A	800 bp	56 °C
***top3A-2*::*TOP3α*-Y342F**	T3A-3-1 / T3A-nt-REV	4600 bp	54 °C
	LB1 / T3A-R3A	800 bp	56 °C
top3A-6	T3A-KO-II-SC-FW1 / T3A-KO-II-WT-REV1	1600 bp	60 °C
	T3A-KO-I-SC-FW / T3A-KO-II-InsDel-REV	850 bp	62 °C
mus81-1	MUS81-1 / MUS?-R2	600 bp	55 °C
	LB1 / MUS?-R2	500 bp	56 °C
recq4A-4	RQ4A-(-2A) / RQ4A-R6A	1000 bp	56 °C
	LB1 / RQ4A-R6A	500 bp	56 °C
