# Supplementary material for: The topoisomerase 3α zinc-finger domain T1 of Arabidopsis thaliana is required for targeting the enzyme activity to Holliday junction-like DNA repair intermediates
Source: PLoS Genet. 2018 Sep 17;14(9):e1007674. doi: 10.1371/journal.pgen.1007674 (PMC6160208; doi:10.1371/journal.pgen.1007674)
Supplement: S6 Table — (PDF) [file pgen.1007674.s017.pdf]

S6 Table: Primer sequences for genotyping.

Primer name	Sequence (5‘-3‘)
**LB1**	GACCATCATACTCATTGCTG
**MUS?-R2**	GCAGCATCAATAAGCTCTTG
**MUS81-1**	AGTGAATCTGATAGTGAGTG
**T3A-3-1**	CATTACCTAGCATGTGTTTC
**T3A-intra-REV**	GACTGTCAGGATTTGACTAG
**T3A-KO-I-Del1-REV-3**	CAACACTGTCACGCGACAT
**T3A-KO-II-InsDel-REV**	ACCGGAGGTCATTAGCAG
**T3A-KO-II-SC-FW-1**	CCTGTAGAGTGAGCTTTGTG
**T3A-KO-II-WT-REV-1**	GACCGGAGCTTGGTACAG
**T3A-KO-II-WT-REV-1**	GACCGGAGCTTGGTACAG
**T3A-KO-I-SC-FW**	CTCCCGACGATACATACG
**T3A-KO-I-WT-REV**	GGGGCCACCGCCTCG
**T3A-nT-REV**	GGATCCACATTAGCTCAC
**T3A-R3A**	GAAAACCGTGTATTAGCATC
**TOPRIM-SC-FW**	CTTTTTATACGCAGGGCTC
**ZnFCCHC1-SC-REV**	CGTTGCTGACAATGTATGC
**ZnFCCHC2-SC-REV**	AGCGTTAGCAAAATGTGAAG
**ZnFGRF-SC-REV**	GTGTTGAGTGATGTAAAAGG
**ZnFT1-SC-REV**	TTCCTGAGTGCCATATCTG
**RQ4A-(-2A)**	GTCCTGATCGTGTTGGACAG
**RQ4A-R6A**	ATCAGAGCCACTCATTGTTG
