# Supplementary material for: The topoisomerase 3α zinc-finger domain T1 of Arabidopsis thaliana is required for targeting the enzyme activity to Holliday junction-like DNA repair intermediates
Source: PLoS Genet. 2018 Sep 17;14(9):e1007674. doi: 10.1371/journal.pgen.1007674 (PMC6160208; doi:10.1371/journal.pgen.1007674)
Supplement: S7 Table — (PDF) [file pgen.1007674.s018.pdf]

S7 Table: Primer combinations for In-Fusion cloning fragment amplification.

Fragment	Primer combination
**Promoter + *TOP3A* 1 bp - 6959 bp + terminator**	T3A-UTR-FW / T3A-Term-REV
**Promoter + *TOP3A* 1 bp - 6959 bp + terminator with overhangs**	T3A-InFu3-FW / T3A-InFu3-REV
**Promoter + *TOP3A* 1 bp - 2027 bp**	T3A-UTR-FW / PuMu2-REV
**Promoter + *TOP3A* 1 bp - 2027 bp with overhangs**	T3A-InFu3-FW / PuMu2-InFu-REV
***TOP3A* 2045 bp - 6959 bp + terminator**	PuMu2-FW / T3A-Term-REV
***TOP3A* 2045 bp - 6959 bp + terminator with overhangs**	PuMu2-InFu-FW / T3A-InFu3-REV
**Promoter + *TOP3A* 1 bp - 141 bp**	T3A-UTR-FW / TOPRIM-REV
**Promoter + *TOP3A* 1 bp - 141 bp with overhangs**	T3A-InFu3-FW / TOPRIM-InFu-REV
***TOP3A* 818 bp - 6959 bp + terminator**	TOPRIM-FW / T3A-Term-REV
***TOP3A* 818 bp - 6959 bp + terminator with overhangs**	TOPRIM-InFu-FW / T3A-InFu3-REV
***TOP3A* 818 bp - 4960 bp**	TOPRIM-FW / N-T3A-REV
***TOP3A* 818 bp - 4960 bp with overhangs**	TOPRIM-InFu-FW / N-T3A-InFu-REV
***TOP3A* 6810 bp - 6959 bp + terminator**	N-T3A-FW / T3A-Term-REV
***TOP3A* 6810 bp - 6959 bp + terminator with overhangs**	N-T3A-InFu-FW / T3A-InFu3-REV
**Promoter + *TOP3A* 1 bp - 4960 bp**	T3A-UTR-FW / N-T3A-REV
**Promoter + *TOP3A* 1 bp - 4960 bp with overhangs**	T3A-InFu3-FW / N-T3A-InFu-REV
**Promoter + *TOP3A* 1 bp - 4982 bp**	T3A-UTR-FW / ZnFT1-REV
**Promoter + *TOP3A* 1 bp - 4982 bp with overhangs**	T3A-InFu3-FW / ZnFT1-InFu-REV
***TOP3A* 5374 bp - 6959 bp + terminator**	ZnFT1-FW / T3A-Term-REV
***TOP3A* 5374 bp - 6959 bp + terminator with overhangs**	ZnFT1-InFu-FW / T3A-InFu3-REV
**Promoter + *TOP3A* 1 bp - 6069 bp**	T3A-UTR-FW / ZnFCCHC1-REV
**Promoter + *TOP3A* 1 bp - 6069 bp with overhangs**	T3A-InFu3-FW / ZnFCCHC1-InFu-REV
***TOP3A* 6121 bp - 6959 bp + terminator**	ZnFCCHC1-FW / T3A-Term-REV
***TOP3A* 6121 bp - 6959 bp + terminator with overhangs**	ZnFCCHC1-InFu-FW / T3A-InFu3-REV
**Promoter + *TOP3A* 1 bp - 6339 bp**	T3A-UTR-FW / ZnFGRF-REV
**Promoter + *TOP3A* 1 bp - 6339 bp with overhangs**	T3A-InFu3-FW / ZnFGRF-InFu-REV
***TOP3A* 6564 bp - 6959 bp + terminator**	ZnFGRF-FW / T3A-Term-REV
***TOP3A* 6564 bp - 6959 bp + terminator with overhangs**	ZnFGRF-InFu-FW / T3A-InFu3-REV
**Promoter + *TOP3A* 1 bp - 6731 bp**	T3A-UTR-FW / ZnFCCHC2-REV
**Promoter + *TOP3A* 1 bp - 6731 bp with overhangs**	T3A-InFu3-FW / ZnFCCHC2-InFu-REV
***TOP3A* 6783 bp - 6959 bp + terminator**	ZnFCCHC2-FW / T3A-Term-REV
***TOP3A* 6783 bp - 6959 bp + terminator with overhangs**	ZnFCCHC2-InFu-FW / T3A-InFu3-REV
