# Supplementary material for: The topoisomerase 3α zinc-finger domain T1 of Arabidopsis thaliana is required for targeting the enzyme activity to Holliday junction-like DNA repair intermediates
Source: PLoS Genet. 2018 Sep 17;14(9):e1007674. doi: 10.1371/journal.pgen.1007674 (PMC6160208; doi:10.1371/journal.pgen.1007674)
Supplement: S8 Table — (PDF) [file pgen.1007674.s019.pdf]

S8 Table: Primer sequences for In-Fusion cloning.

Primer name	Sequence (5‘-3‘)
**N-T3A-FW**	TAAGTCTACTTACTACGAACTT
**N-T3A-InFu-FW**	ACTGATGAGAGTGAATAAGTCTACTTACTACGAACTT
**N-T3A-InFu-REV**	TAGTAAGTAGACTTATTCACTCTCATCAGTGTTATTA
**N-T3A-REV**	TTCACTCTCATCAGTGTTATTA
**PuMu2-FW**	GGAGACCGATTCTTTTTCG
**PuMu2-InFu-FW**	ATCAGCTTTCCTCGTACGGAGACCGATTCTTTTTCG
**PuMu2-InFu-REV**	GTACGAGGAAAGCTGATGAAACCAGCCTGATATAATTC
**PuMu2-REV**	GAAACCAGCCTGATATAATTC
**T3A-InFu3-FW**	AATCGAATTCAAGCTTGATTGTACAAGCCTGACCA
**T3A-InFu3-REV**	ACGCGTCCGCGGTTAATTAAGTGGAAACTATTCAGTGATTC
**T3A-Term-REV**	GTGGAAACTATTCAGTGATTC
**T3A-UTR-FW**	GATTGTACAAGCCTGACCA
**TOPRIM-FW**	TTCTCTGCGTTAATTGACAG
**TOPRIM-InFu-FW**	GGCGGTGGCCCCGTGTTCTCTGCGTTAATTGACAG
**TOPRIM-InFu-REV**	AATTAACGCAGAGAACACGGGGCCACCGCC
**TOPRIM-REV**	CACGGGGCCACCGCC
**ZnFCCHC1-FW**	CGGGTTCCAGCATCTCG
**ZnFCCHC1-InFu-FW**	ACAAGACAGAGTAATCGGGTTCCAGCATCTCG
**ZnFCCHC1-InFu-REV**	AGATGCTGGAACCCGATTACTCTGTCTTGTGTTGC
**ZnFCCHC1-REV**	ATTACTCTGTCTTGTGTTGC
**ZnFCCHC2-FW**	CGTAATAACTCTAACGGTAAC
**ZnFCCHC2-InFu-FW**	CCAGTGAGTGGTATACGTAATAACTCTAACGGTAAC
**ZnFCCHC2-InFu-REV**	GTTAGAGTTATTACGTATACCACTCACTGGCTC
**ZnFCCHC2-REV**	TATACCACTCACTGGCTC
**ZnFGRF-FW**	ATAAACAACAGTAGTGGGAA
**ZnFGRF-REV**	TGTACTTTCATCTGCATTATAA
**ZnFT1-FW**	GGACCGACATTGGAAGCA
**ZnFT1-InFu-FW**	ACAGCTGGTGAAGTTGGACCGACATTGGAAGCA
**ZnFT1-InFu-REV**	TTCCAATGTCGGTCCAACTTCACCAGCTGTCTG
**ZnFT1-REV**	AACTTCACCAGCTGTCTG
