# Supplementary material for: The topoisomerase 3α zinc-finger domain T1 of Arabidopsis thaliana is required for targeting the enzyme activity to Holliday junction-like DNA repair intermediates
Source: PLoS Genet. 2018 Sep 17;14(9):e1007674. doi: 10.1371/journal.pgen.1007674 (PMC6160208; doi:10.1371/journal.pgen.1007674)
Supplement: S1 Method — (PDF) [file pgen.1007674.s020.pdf]

S1 Method: qRT-PCR analysis of *HEI10* and *MRD1* gene expression

For the analysis of *HEI10* and *MRD1* gene expression in complementation lines, quantitative Real-Time PCR was performed. RNA from two-week-old plantlets was extracted using the RNeasy Plant Mini Kit (Qiagen GmbH, Hilden, Germany). Reverse-transcription of RNA into cDNA was performed using an oligo-$(dT)_{18}$ primer and the RevertAid First Strand cDNA Synthesis Kit (Thermo Fisher Scientific GmbH, Waltham, MA, USA). For the qRT-PCR analysis, the LightCycler 480 KAPA SYBR Fast Mastermix (Sigma-Aldrich, Steinheim, Germany) was used in total reaction volumes of 20 µl (10 µl KAPA SYBR Fast Mastermix, 0.08 µl of each primer [50 µM], 4.84 µl ddH_2O and 5 µl cDNA template). The analysis was performed on a Light Cycler 480 (Roche Diagnostics, Mannheim, Germany) with an initial denaturation at 95 °C for 3 min followed by 50 cycles of amplification (95 °C/10s, 60 °C/20s, 72 °C/1s) and a terminal melting curve from 65 °C – 97 °C. Data was normalized by amplification of the housekeeping genes *ACTIN2* (At3g18780) and At4g34270. Following primer pairs were used for amplification: *ACTIN2*: Actin2-FW 5'-CAGATGCCCAGAAGTCTTG-3' and Actin2-REV 5'-GTGCTGTGATTTCTTTGCTC-3'. At4g34270: At4g34270-F1 5'-AGATGAACTGGCTGACAATG-3' and At4g34270-R1 5'-TGTTGCTTCTCTCCAACAGT-3'. *HEI10* 5' end: HEI10-F1 5'-TGTTCTTCAATCACTTTCTGC-3' and HEI10-R1 5'-TCATTACTGAGAATCTTGCTG-3'. *HEI10* 3' end: HEI10-F2 5'-TCCAACCAGCAAACAACTTC-3' and HEI10-R2 5'-GGTGAGTCGGTGGAGATG-3'. *MRD1* 5' end: MRD1-F1 5'-CCTAAAACGGACATACTAATC-3' and MRD1-R1 5'-GCAGATTTACATGATTCAAGG-3'. *MRD1* 3' end: MRD1-F2 5'-TGTTTTCGTGTCATTGGGTG-3' and MRD1-R2 5'-GCTCAACATCAACTTTCCAC-3'.
