# Supplementary material for: The topoisomerase 3α zinc-finger domain T1 of Arabidopsis thaliana is required for targeting the enzyme activity to Holliday junction-like DNA repair intermediates
Source: PLoS Genet. 2018 Sep 17;14(9):e1007674. doi: 10.1371/journal.pgen.1007674 (PMC6160208; doi:10.1371/journal.pgen.1007674)
Supplement: S2 Method — (PDF) [file pgen.1007674.s021.pdf]

S2 Method: 5' RACE analysis of *HEI10* cDNA

For the analysis of 5' *HEI10* cDNA ends a 5' RACE (5' rapid amplification of cDNA ends) PCR was performed. Therefore, RNA was extracted from two-week-old wild type plantlets using the RNeasy Plant Mini Kit (Qiagen GmbH, Hilden, Germany). RNA was reverse-transcribed into cDNA using the *HEI10* gene-specific primer Hei10-GSP1 5'-ACACCTCTTGCCCATCTTC-3' and the RevertAid First Strand cDNA Synthesis Kit (Thermo Fisher Scientific GmbH, Waltham, MA, USA). For RNA degradation, a mixture of 1:1 RNaseH and RNaseT1 (Thermo Fisher Scientific GmbH) was added, incubated at 37 °C for 30 min, followed by a DNA purification with the High Pure PCR Product Purification Kit (Roche Diagnostics, Mannheim, Germany). Using a terminal deoxynucleotidyl transferase (Thermo Fisher Scientific GmbH), a poly-C tail was added. In a PCR reaction using the *HEI10* gene-specific primer Hei10-GSP2 5'-GATATGCTGTATGGACCTGCTC-3' and an oligo-dG primer with additional overhang 5'-GGCCACGCGTCGACTAGTACGGGGGGGGGGGGG-3', the tailed cDNA was amplified. Afterwards, a nested PCR was performed using the *HEI10* gene-specific primer Hei10-GSP3 5'-TTGCATACCCTCACATTTCTGAC-3' and the overhang-specific primer 5'-GGCCACGCGTCGACTAGTAC-3'. The obtained PCR product was purified and sequenced by Sanger sequencing.
